# Identification of gene conversion events in horse IGHV suggests preferential hotspots for diversification

**DOI:** 10.1007/s00251-026-01400-7

**Published:** 2026-05-19

**Authors:** Juliana Edelvacy Lima Pinto, João Henrique Brandão Gervásio, Joseph Chi-fung Ng, Adriano Gomes-Silva, Herbert L. de Matos Guedes, Luiz Cunha, Leda R. Castilho, Jerson Lima da Silva, Glória Regina Franco, Carlena Navas, Liza Figueiredo Felicori

**Affiliations:** 1 Laboratory of Synthetic Biology and Biomimetics, Department of Biochemistry and Immunology, Institute of Biological Sciences (ICB), Federal University of Minas Gerais, Belo Horizonte, MG Brazil; 2 Model-Based Evolutionary Genomic Unit, Okinawa Institute of Science and Technology (OIST), Tancha, 904-0412 Okinawa Japan; 3 Research Department of Structural and Molecular Biology, University College London, London, WC1E 6BT UK; 4 Interdisciplinary Laboratory of Medical Research, Oswaldo Cruz Institute, Oswaldo Cruz Foundation, Rio de Janeiro, RJ Brazil; 5 Mycobacteriosis Clinical Research Laboratory, Evandro Chagas National Institute of Infectious Diseases, Oswaldo Cruz Foundation, Rio de Janeiro, RJ Brazil; 6 Laboratory of Immunobiotechnology, Department of Immunology, Paulo de Góes Institute of Microbiology, Federal University of Rio de Janeiro, Rio de Janeiro, RJ Brazil; 7 Laboratory of Clinical Immunology, Oswaldo Cruz Institute, Oswaldo Cruz Foundation, Rio de Janeiro, RJ Brazil; 8 Vital Brazil Institute, Niterói, RJ Brazil; 9 Cell Culture Engineering Laboratory, COPPE, Federal University of Rio de Janeiro, Rio de Janeiro, RJ Brazil; 10 Institute of Medical Biochemistry Leopoldo de Meis, Federal University of Rio de Janeiro, Rio de Janeiro, RJ Brazil; 11 Laboratory of Biochemical Genetics, Department of Biochemistry and Immunology, Institute of Biological Sciences (ICB), Federal University of Minas Gerais, Belo Horizonte, MG 30161-970 Brazil

**Keywords:** Gene Conversion, Immunoglobulin, Antibody Repertoire, Diversity, Pseudogenes

## Abstract

The humoral immune response relies on a diverse antibody repertoire, which is expanded through processes such as somatic hypermutation, class-switch recombination and gene conversion. These processes are primarily mediated by activation-induced cytidine deaminase (AID). Gene conversion generates diversity in immunoglobulin heavy and light chains (IGHVs) in species such as chickens and rabbits, though it has not been widely studied. Since 80% of the equine IGHV repertoire originates from only three functional gene segments, we examined gene conversion events in horses to assess their role in antibody diversification. Using a modified version of BrepConvert, which optimized analysis time, we identified gene conversion events in 6.9% of immunoglobulin sequences. The results showed a local preference, with most events occurring at the beginning of framework region 1 (FR1) and within complementarity-determining region 2 (CDR2). Pseudogenes IGHV4-35, IGHV4-53, and IGHV4-38 were utilized most frequently, while functional genes IGHV4-21, IGHV4-22, and IGHV4-29 exhibited the highest event frequencies. Interestingly, while most mismatched regions were only three nucleotides long, 91% of these events are flanked by specific sequences (six nucleotides at the 5′ end and one nucleotide at the 3′ end). Furthermore, functional pseudogene pairs often share identical leader regions of 5–26 nucleotides, suggesting expanded events. We also identified a potential association between these events and local non-B DNA conformations, as well as with the zinc finger protein ZNF691, which supports the involvement of DNA-binding factors. Together, these findings demonstrate that gene conversion significantly contributes to equine antibody diversity by targeting specific IGHV regions.

**Supplementary Information:**

The online version contains supplementary material available at 10.1007/s00251-026-01400-7.

## Introduction

In the late 19th century (1890), Behring and Kitasato pioneered the use of serum from immunized animals, rats and horses, to develop antitoxins against diphtheria and tetanus, respectively (Behring 1890). In 1891, Behring achieved the first successful treatment of a child with diphtheria. This breakthrough led Behring to receive the inaugural Nobel Prize in Physiology or Medicine in 1901 (Grundbacher 1992; Walther et al. 2015). Since then, horses have remained indispensable for antivenom production and have also been used during the COVID-19 pandemic for therapeutic serum production in countries such as Argentina (Zylberman et al. 2020). Their extensive use is largely owing to their high blood volume, which allows repeated antibody harvesting for therapeutic applications (Manteca Vilanova et al. 2019). Nevertheless, despite their long-standing biotechnological importance, studies focusing on equine immunoglobulin repertoire and genomic organization remain limited.

Regarding the immunoglobulin locus organization of this species, it is currently known that the equine immunoglobulin heavy chain (IGH) locus is located on chromosome 24 and contains 104 IGHV (variable) genes, grouped into 7 subgroups, 44 IGHD genes, 9 IGHJ genes, and 11 IGHC (constant) genes. Of the 104 variable genes, only 21 IGHV genes are functional, along with 9 open reading frames (ORFs) and 74 pseudogenes (Lefranc et al. 2015; Wibmer and Mashilo 2022). Interestingly, pseudogenes outnumbered in three fold functional genes at the equine IGH locus. Previous work from our group showed that only three functional genes were used in more than 80% of horse IGHV repertoires, suggesting that gene rearrangement is not the primary mechanism for generating diversity in this species (Navas et al. 2022). Studies in different species indicate that pseudogenes may contribute to the generation of diversity in the antibody repertoire through mechanisms such as gene conversion or non-canonical recombination (Meyer et al. 1997). Given the fact that the equine genome has a substantially higher number of pseudogenes compared to functional immunoglobulin genes, it is relevant to investigate whether these elements also partake in the diversification of the antibody repertoire in horses.

Pseudogenes (PGs) are genomic sequences that resemble functional genes, but have altered or lost functionality and are present across all life forms (Cheetham et al. 2020). Although their biological relevance has long been debated, increasing evidence supports functional roles (McCarrey 1987; Charrier et al. 2012; Chiang et al. 2018). One function particularly relevant for immunoglobulins is the transfer of PG fragments to their parental genes through non-allelic recombination, known as gene conversion (Bischof et al. 2006). This mechanism has been shown to be the primary way in which antibody diversity is generated in chickens and rabbits by integrating PG fragments into recombined V segments of immunoglobulin heavy and light chains (Tang and Martin 2007; Becker and Knight 1990; Reynaud et al. 1987). Although gene conversion mechanisms are not fully understood, they have been shown to depend on the activation-induced cytidine deaminase (AID) (Muramatsu et al. 2000; Harris et al. 2002). AID deletion results in the cessation of three main processes of immunoglobulin diversification: somatic hypermutation, class switch recombination, and gene conversion (Muramatsu et al. 2000; Revy et al. 2000; Arakawa et al. 2002; Harris et al. 2002). Even though all three processes begin with an AID-induced lesion, only during gene conversion homologous recombination-mediated repair occurs (Seo et al. 2024).

Previous studies suggest that species in which gene conversion is the primary mechanism of diversification have limited functional gene segments or restricted usage of them (Duvvuri and Wu 2012). This is the case for chickens, which have only one functional immunoglobulin light chain family and use 25 upstream pseudogenes as templates for recombination (Reynaud et al. 1987), and rabbits, which preferentially use one VH gene segment (VH1) in 80–90% of rearrangements and use other VH pseudogene segments as templates for gene conversion (Becker and Knight 1990). Although gene conversion has been well documented in chickens and rabbits, its occurrence in equine immunoglobulins is not thoroughly described, even though horses have the necessary machinery for gene conversion and the restricted use of IGHV gene segments (Clegg 1987; Navas et al. 2022).

Furthermore, significant efforts have been devoted to estimating gene conversion events using various methods, including laboratory strains (Hilliker et al. 1994), human genomic analysis (Kong et al. 2002) and animal models (Johnston et al. 2016). However, the high cost and extensive data required to reach statistical significance often affect these direct approaches. Consequently, indirect computational methods have emerged as a cost-effective way of inferring recombination from sequenced genomes. Despite these advances, identifying gene conversion in antibody repertoires remains challenging. Accurate detection requires extensive sampling to capture rare events, resulting in large datasets that exceed the capacity of traditional algorithms. Furthermore, inconsistencies reported across software packages (Mansai et al. 2010) highlight the need for robust, specialised and scalable workflows.

To address these limitations, we have implemented an optimised version of BrepConvert to scale up the identification of gene conversion in annotated IGHV sequences. Despite the prevalence of pseudogenes and limited IGHV usage in horses, the role of gene conversion in equine antibody diversification remains unclear. The aim of this study is to identify and quantify these events in the equine repertoire. Elucidating this mechanism could reveal new pathways for antibody diversity, with potential applications in vaccine development and therapeutics, similar to advancements observed in avian models (Seo et al. 2024).

## Results

### Identification of gene conversion events in the equine antibody repertoire

To identify gene conversion events in the equine antibody repertoire, we sequenced the IGHV mRNA repertoire of four horses and analyzed transcriptomics data from an additional four horses previously sequenced by our group, as described by Navas et al. (2022). For each animal, an average of 286,538 reads were obtained, yielding 94,280 high-quality annotated sequences and 67,643 unique sequences (see Table 1). The sequences were then processed using BrepConvert (Mallaby et al. 2023) to identify gene conversion events. This package was designed to analyze long-read sequencing data of a lower throughput than our approach here. To further improve efficiency of the BrepConvert workflow, we added pBlat (Wang and Kong 2019) to the pipeline for faster and more efficient sequence alignment, along with additional modifications to the functional gene parsing and alignment with repertoire steps. Additionally, we excluded pseudogenes that were not located upstream of functional genes, as evidenced in IGHV in different organisms suggesting that gene conversion occurs using pseudogenes positioned 5′ of their corresponding functional counterparts (Reynaud et al. 1989; Becker and Knight 1990; Walther et al. 2016).

Through the modifications, a significant reduction in runtime was observed for the identification of gene conversion events. Specifically, processing 1,000 randomly selected annotated sequences from horse repertoires using the modified BrepConvert required 44 min on an eight-core processor, a substantial improvement over the approximately 5 h required by the original version (Supplementary Fig. 1). This dramatic decrease in analysis time enabled a comprehensive investigation of gene conversion events in horse IGHV. We observed that on average 4,402 sequences per horse were identified as susceptible to gene conversion, representing 6.90% of the total unique sequences analyzed (Table 1). These results demonstrate that the modified version of BrepConvert enables the identification of a significant proportion of the IGHV horse repertoire that is susceptible to gene conversion.

**Table 1:** Number of Gene Conversion Events Identified through Brepconvert in Horse IGHV

Horse	Raw	Pre-proc	PS	Annotation	Unique Seqs	BrepConvert	Filter	% GC
1	94,501	91,830	38,430	38,430	33,984	5,158	2,653	7.80
2	222,339	216,902	75,118	75,118	62,435	7,379	3,730	5.98
3	452,715	444,022	202,685	202,685	132,912	16,012	8,042	6.05
4	133,538	131,481	57,447	57,447	51,791	6,741	3,141	6.07
5	210,964	203,754	89,070	89,070	65,152	11,612	6,493	9.97
6	288,611	278,484	111,060	111,060	73,434	11,591	6,035	8.21
7	408,483	341,226	26,064	26,064	18,078	2,794	1,342	7.42
8	481,150	456,456	154,359	154,359	103,354	8,869	3,780	3.66
Mean	286,538	270,520	94,280	94,280	67,643	8,770	4,402	6.90
Total	2,292,301	2,164,155	754,233	754,233	541,140	70,156	35,216	

*Raw*: Raw data; *Pre-proc*: Pre-processing; *PS*: Phred Score Filter; *Filter*: Gene Order Filter; *% GC*: Percentage of gene conversion events (Percentage calculated on Unique Seqs). Horses 5–8 were obtained from the work of Navas et al. (2022)

### Characteristics of mismatched regions associated with gene conversion events in equine antibody IGHV genes.

**Fig. 1:**
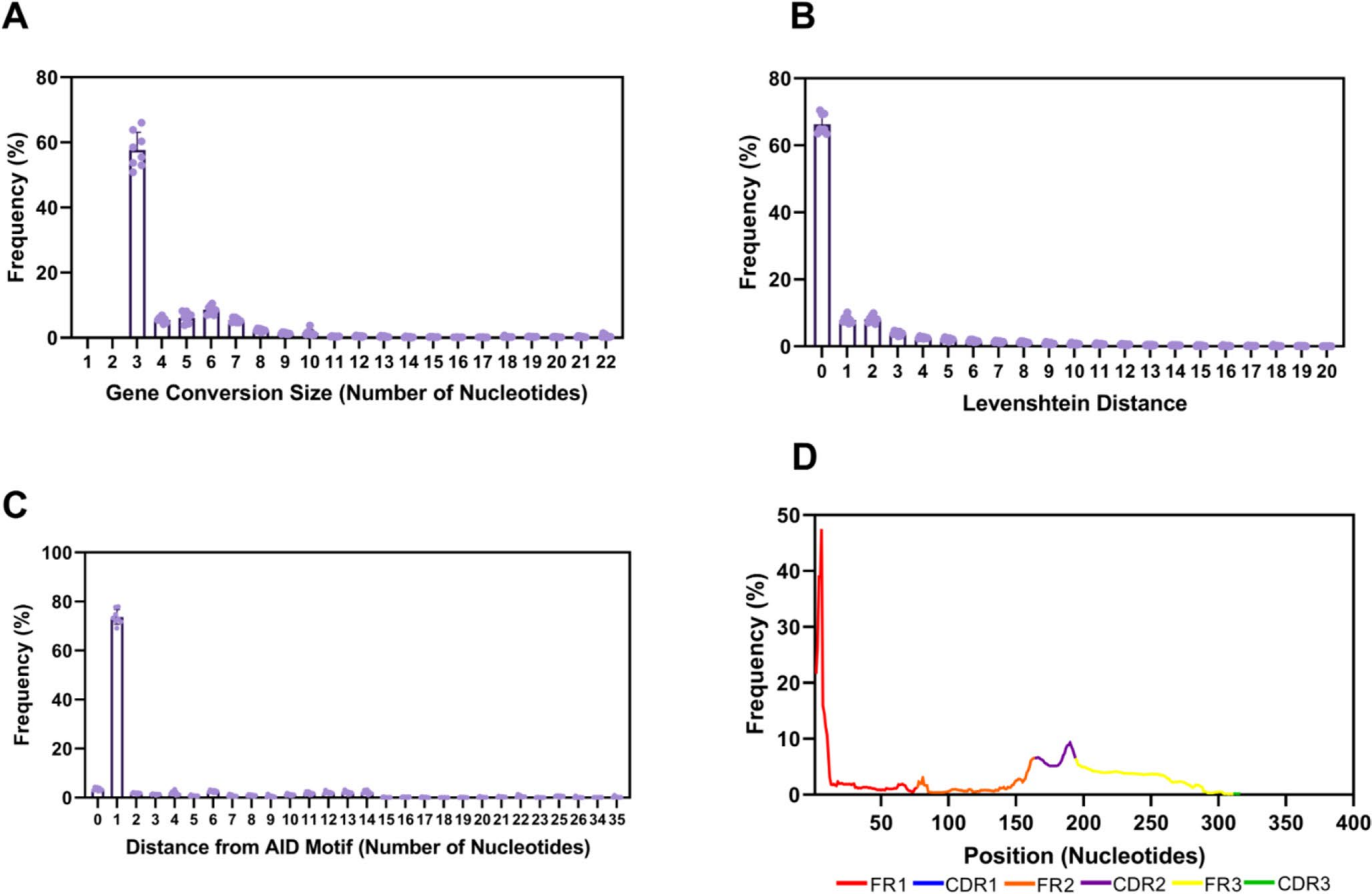
Features of mismatched regions associated with gene conversion events in IGHV genes. (**A**) Number of nucleotide mismatches between repertoire IGHV genes and their corresponding germline functional genes, as identified by BrepConvert. (**B**) Levenshtein distance between each mismatched region and its closest pseudogene, reflecting the sequence similarity between candidate donor pseudogenes and repertoire IGHV genes. (**C**) Distance (in nucleotides) between each mismatched region and the nearest AID motif, indicating the spatial relationship between these regions and AID targeting sites. (**D**) Distribution of mismatched regions across the variable domains of the immunoglobulin heavy chain. Colored traces indicate the positions of these regions within each V-gene segment

To better understand the patterns associated with gene conversion in horse IGHV genes, we used Brepconvert to analyze the properties of mismatched regions identified as candidate gene conversion segments. These regions were defined as contiguous stretches of mismatches between repertoire and functional germline genes that are similar to pseudogene sequences. The length of these mismatched regions ranged from 3 to 251 nucleotides (Supplementary Table 1), with approximately 60% being 3 nucleotides long (Fig. 1A). Mismatched regions longer than 10 nucleotides accounted for 13% of the total, while those exceeding 100 nucleotides represented only 1% (Supplementary Fig. 2). Although this suggests that most events are short, it is important to emphasize that these measurements correspond only to the mismatched regions and may not reflect the full extent of the underlying gene conversion events.

To further assess sequence similarity, we calculated the Levenshtein distance between each mismatched region and its closest pseudogene. Approximately 60% of sequences exhibited zero distance, reflecting complete identity with their corresponding pseudogenes (Fig. 1B). Notably, the sequences with length of three nucleotides showed a Levenshtein distance of zero, indicating that nearly all of these short, mismatched three-nucleotide sequences are identical to the candidate donor pseudogene.

Given the role of AID in generating gene conversion (Harris et al. 2002), we assessed the proximity of mismatched regions to AID motifs. Approximately 74% of gene conversion events predicted by BrepConvert were located near an AID motif, typically one nucleotide away (Fig. 1C). In most cases, mismatches were positioned adjacent to, rather than directly within, AID target motifs. In addition, AID motifs closest to these mismatched regions were predominantly located in the Framework 1 (FR1) region of IGHV genes (Fig. 1D, Supplementary Fig. 3).

#### Limited overlap between AID hotspots and gene conversion–associated mismatches in equine antibody IGHV genes

Given that most identified gene conversion–associated mismatches were three nucleotides in length, we considered whether these regions might instead reflect clustered somatic hypermutation (SHM) events. Previous studies have shown that SHM in immunoglobulin variable regions predominantly occurs as single-nucleotide substitutions within AID hotspot motifs, such as WRCY (Bransteitter et al. 2004; Di Noia and Neuberger 2007). Although SHM is typically characterized by single-nucleotide changes, rare multi-nucleotide mutations can occur at low frequency and may be mistaken for gene conversion events (Wilson et al. 1998; Pilzecker and Jacobs 2019; Sepúlveda et al. 2022).

However, SHM events are generally confined to AID hotspot motifs, whereas gene conversion–associated mismatches are not restricted to these regions. To exclude the possibility that the mismatches identified here were misclassified SHM events, we therefore assessed their overlap with AID hotspot motifs. Analysis of 2,332,592 AID hotspot positions revealed that 94.4% (*n* = 2,202,584) did not overlap with putative gene conversion sites. Partial overlaps were observed in 5.6% (*n* = 129,698) of cases, whereas complete containment of mismatched regions within hotspot boundaries was rare (0.01%, *n* = 310). Together, these results indicate that the vast majority of mismatched regions occur outside AID hotspots, supporting their classification as gene conversion events.

#### Preferential involvement of specific functional genes and pseudogenes in regions associated with gene conversion events

**Fig. 2:**
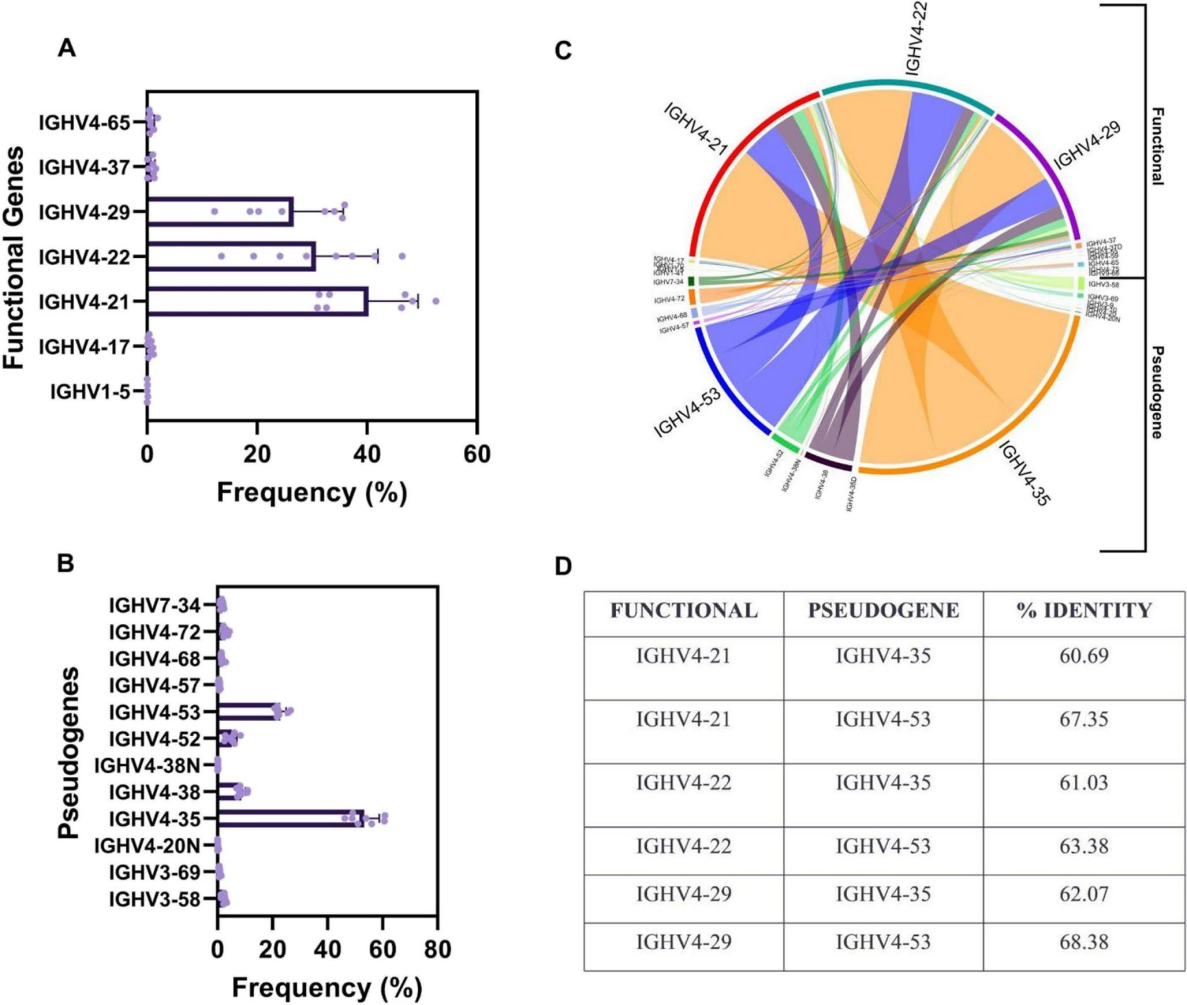
Analysis of the most common functional and pseudogenes IGHV used for equine gene conversion. (**A**) Frequency of functional gene families undergoing gene conversion. (**B**) Frequency of pseudogene families undergoing gene conversion. (**C**) Chord Diagram showing the rate of occurrence of gene conversion events between the pseudogenes and functional genes. The larger the bar, the greater is the contribution of the gene to the gene conversion event. (**D**) Table showing the identity percentage between the most used functional and pseudogenes for gene conversion

After characterizing the general features of mismatched regions associated with gene conversion events in equine IGHV, we next examined which gene families and specific functional genes and pseudogenes were most frequently involved. The IGHV4 family, the largest IGHV family in horses, was the most used for both functional genes (Fig. 2A) and pseudogenes (Fig. 2B). Among functional genes, IGHV4-21 exhibited the highest number of gene conversion events, followed by IGHV4-22 and IGHV4-29 (Fig. 2A), which are also the most frequently used IGHV genes in the equine repertoire (Navas et al. 2022). The pseudogenes with highest frequency of contribution to gene conversion events were IGHV4-35, IGHV4-53, and IGHV4-38 (Fig. 2B).

Analysis of event frequency revealed that only a small subset of functional genes served as major targets for gene conversion (Fig. 2C). Notably, IGHV4-21 experienced the highest number of conversions originating from pseudogene IGHV4-35, while IGHV4-22 showed the most events involving pseudogenes IGHV4-35 and IGHV4-53. However, the sequence identity between these functional genes and pseudogenes ranged from 60% to 68% (Fig. 2D). This contrasts with the classical expectation, supported by early literature, that immunoglobulin gene conversion preferentially occurs between sequences sharing greater than 80% identity (McCormack and Thompson 1990).

### Evidence for extended gene conversion beyond observed mismatches

**Fig. 3:**
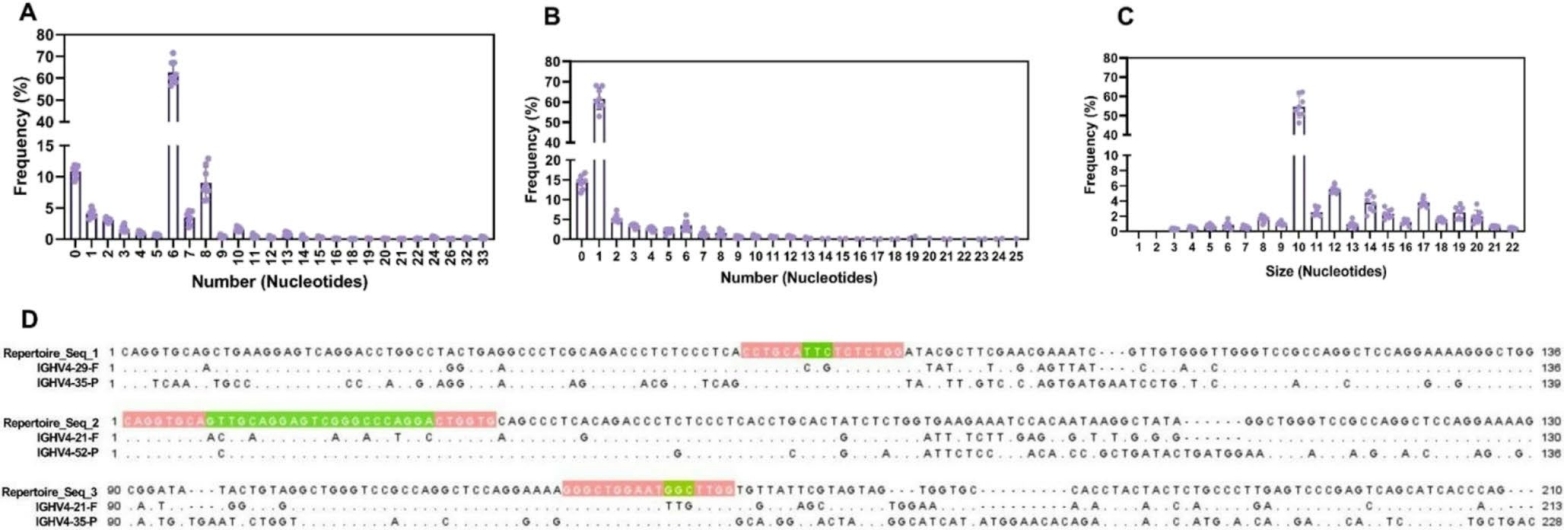
Size of identical regions between functional genes and pseudogenes flanking the gene conversion mismatched region. (**A**) The size of identical regions 5’ from the gene conversion events. (**B**) Size of identical regions 3’ from the gene conversion events. (**C**) Size of expanded gene conversion events, combining the lengths at 5’ and 3’ from the event. (**D**) Alignment of representative equine IGHV repertoire sequences with the corresponding functional germline gene (**F**) and potential donor pseudogenes (P). From top to bottom, the sequences shown are the repertoire sequence, the functional germline and the candidate pseudogene donors. Gene conversion events are highlighted in green and expanded gene conversion events, including 5’ and 3’ identical lengths, are highlighted in light red. Dots indicate positions that are identical to the repertoire sequence

In order to investigate how gene conversion occurs between functional genes and pseudogenes with relatively low sequence identity, we examined the 5’ and 3’ flanking regions of converted events. Previous work by McCormack and Thompson (1990) observed that gene conversion is usually initiated at the 5’ end in a region of sequence homology and that most of these events are flanked on the 5’ and 3’ sides by blocks of nucleotide sequences of variable lengths at which the functional gene segment and the pseudogene donor segment are identical. In our study, we verified the presence of conserved sequences, mostly consisting of six to eight nucleotides, upstream (5’) of the region of mismatch with the functional gene and the pseudogene region (Fig. 3A). Additionally, the downstream region (3’) of these mismatches presented one nucleotide identical to the pseudogene in around 60% of cases (Fig. 3B). Interestingly, 91% of events measuring three nucleotides in size were flanked by these six-nucleotide identical regions at the 5’ end and one nucleotide at the 3’ end.

This pattern of local sequence identity suggests that the true extent of the gene conversion event may exceed the observed mismatched region. For example, events with a mismatch of three nucleotides (Fig. 1A) may extend to approximately 10–12 nucleotides when including the conserved flanking regions (Fig. 3C). The Fig. 3D exemplifies the presence of these identical regions, in mismatched regions of three nucleotides in size (highlighted in green), the sequences are predominantly identical to the pseudogene, yet differ from the functional gene. Meanwhile, the upstream (5′) and downstream (3’) region (highlighted in light red) shows high similarity to both sequences. Similar patterns are observed in longer mismatched regions (≥ 10 nucleotides), supporting the idea that these flanking regions may be extensions of the original gene conversion event.

Furthermore, McCormack and Thompson (1990) suggested that gene conversion events, beyond the initially identified mismatched regions, could extend to the leader region of the light chain of chicken immunoglobulins, when additional stretches of sequence homology are present between donor and acceptor sequences. Consistent with this, our study reveals similar patterns in equine IGHV (Supplementary Fig. 4). Alignments between frequently used functional genes and pseudogenes show conserved sequences at the end of the leader region (highlighted in light red), ranging from 5 to 26 nucleotides. This upstream homology suggests that gene conversion events may extend beyond the detected mismatched regions into the leader sequence, reaching lengths of 15 to 36 nucleotides in most cases. Therefore, the length of gene conversion events identified in this study is likely underestimated, and may be larger than indicated by the mismatched regions alone.

### Non–B DNA–forming motifs are enriched and localized near gene conversion hotspots

**Fig. 4:**
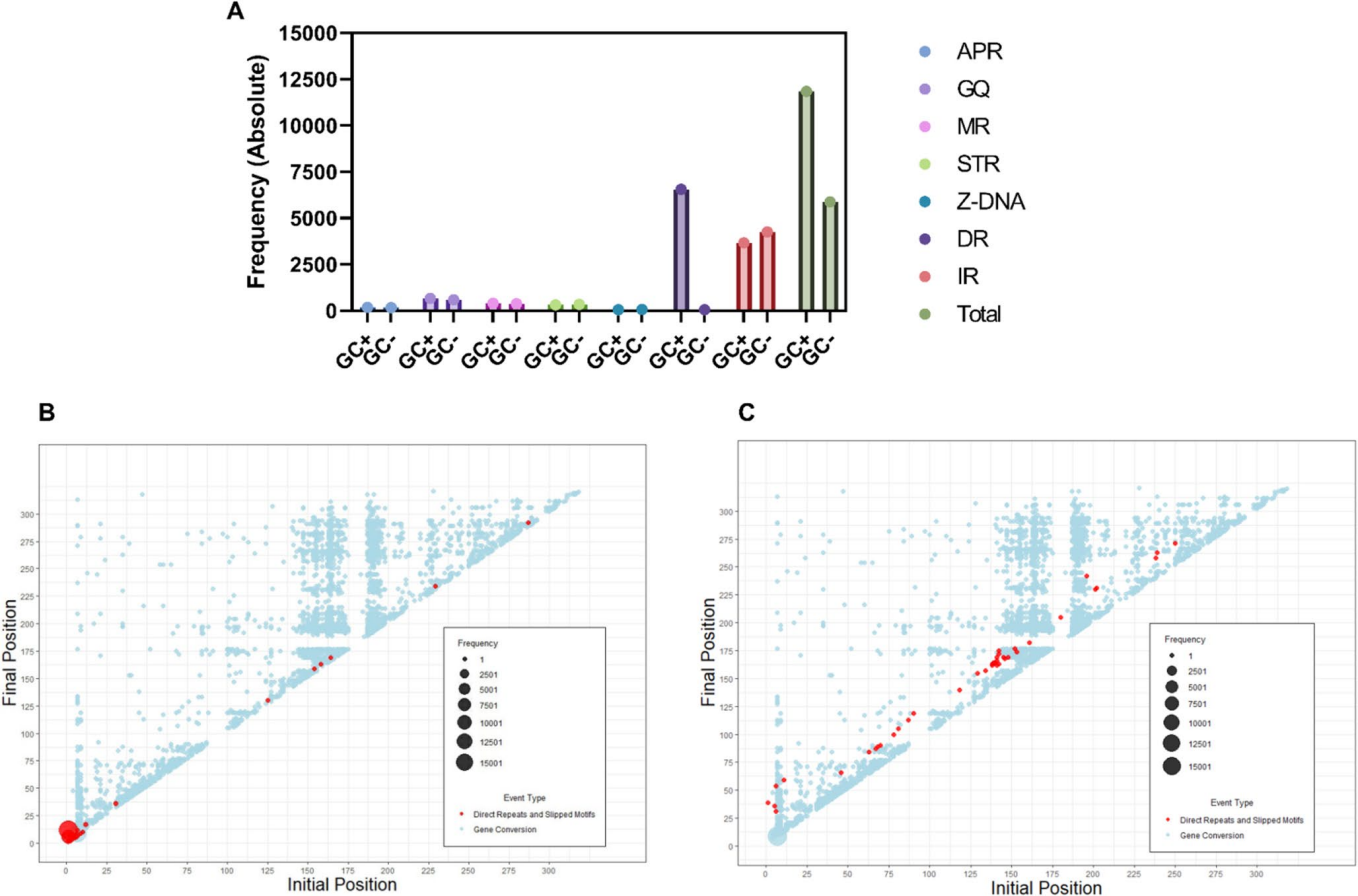
Identification of non-B DNA motifs in sequences associated with gene conversion events. (**A**) Frequency of non-B DNA motifs in sequences with identifiable gene conversion events (GC+) and in sequences without identifiable events (GC–). Motifs include: APR, A-phased repeat; GQ, G-quadruplex–forming repeat; MR, Mirror Repeat; STRs, Short Tandem Repeats; Z-DNA, Z-DNA motif; DR, Direct Repeats and Slipped Motifs; IR, Inverted Repeat; (**B**) Positions of Direct Repeats and Slipped Motifs in GC+ sequences relative to gene conversion events. (**C**) Positions of Direct Repeats and Slipped Motifs in GC– sequences relative to gene conversion events

Beyond local sequence similarity, previous studies have highlighted the role of non–B DNA conformations in facilitating gene conversion (Chuzhanova et al. 2009; Bacolla et al. 2018). To evaluate this in horses, we assessed the presence of non–B DNA forming motifs in regions with identified gene conversion events. As shown in Fig. 4A, sequences containing gene conversion events (GC+) displayed a higher frequency of Direct Repeats and Slipped motifs (DR) compared with sequences lacking identifiable events (GC–). Examining the positional distribution of these motifs (Fig. 4B), we found that they were concentrated near the beginning of the V region in sequences with identified gene conversion events, which corresponds to the area with the highest density of these events (Fig. 1D). In contrast, this enrichment was not observed in sequences without gene conversion events (Fig. 4C).

### Identification of motifs in regions flanking expanded gene conversion events

Finally, we assessed whether equine IGHV gene conversion events occur more frequently within specific sequence contexts. Since the size of the identified events could be larger, as demonstrated in Fig. 3, we analyzed whether the conservation of flanking 5′ and 3′ regions from expanded gene conversion events could suggest preferred sites in IGHV sequences for these events to occur. However, we did not perform 5′ upstream motif analysis since more than half of the expanded gene conversion event 5′ from the mismatched region originated from nucleotide 1 (approximately 62%).

In contrast, a conserved motif was identified downstream (3′) of the expanded gene conversion sites (Supplementary Fig. 5). The motif (AAGGAGTC) was present in approximately 60% of the analyzed sequences. Comparison with databases of transcription factor binding sites revealed similarity to a predicted binding site for Zinc Finger 691 (ZNF691) (Table 2). Together, these results indicate that gene conversion events occur in equine IGHV genes and are associated with preferred sequence features within the 3’ region of this gene segment.

**Table 2:** Sequence motifs downstream of expanded gene conversion events and predicted binding sites

Horse	Motif	*P*-Value	Frequency (%)	Binding Site	*P*-Value
1	AAGGAGTC	1.2e-006	57.0	ZNF691	2.28e-04
2	AAGGAGTC	8.1e-009	55.6	ZNF691	2.27e-04
3	AAGGAGAC	4.5e-024	52.8	Not found	-
4	AGGAGTCA	1.8e-011	51.0	ZNF691	4.55e-04
5	AAGGAGTC	3.5e-026	63.5	ZNF691	2.23e-04
6	AAGGAGTCA	2.9e-013	47.4	ZNF691	4.49e-04
7	AAGGAGTC	1.1e-008	70.6	ZNF691	1.97e-04
8	AAGGAGTC	1.3e-007	52.1	ZNF691	2.21e-04

## Methods

### Horse PBMC samples

**Fig. 5:**
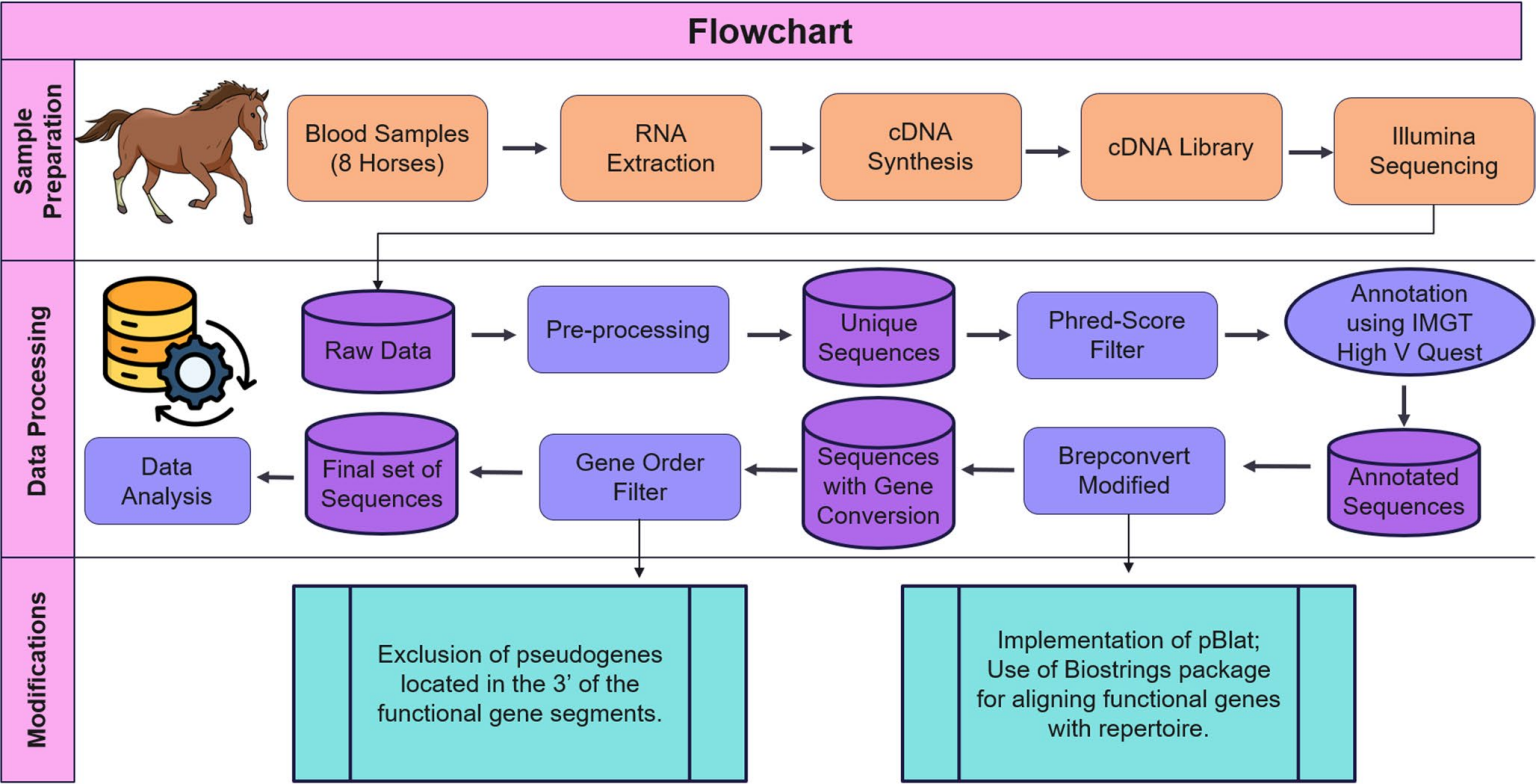
Workflow for identifying gene conversion events in the equine IGHV repertoire. The analysis was divided into three main stages: (1) sample preparation; (2) data processing and detection of gene conversion events; and (3) methodological optimizations implemented to improve the efficiency and accuracy of gene conversion identification. DB: Data Bank

Peripheral blood samples were obtained from eight healthy mixed-breed horses (male and female, 3–9 years old) represented in Fig. 5. Four samples originated from the study by Navas et al. (2022), and the remaining four were collected and processed under the same conditions for the present work. The animals used in Navas et al. (2022) were provided through a partnership with the Center for Research and Production of Immunobiologicals (CPPI), State of Paraná, while the additional four samples were obtained in collaboration with Instituto Vital Brazil, State of Rio de Janeiro. Laboratory analyses were performed to rule out diseases such as leptospirosis, brucellosis, equine babesiosis, glanders, and equine infectious anemia, as previously described by Navas et al. (2022) and Silva et al. (2025). All horses were treated with dewormers and antirabies vaccines before sample collection.Approximately 35 ml of peripheral blood was obtained from each animal using Vacutainer tubes containing sodium heparin anticoagulant (BD Vacutainer). Peripheral blood mononuclear cells (PBMCs) were isolated by centrifugation on a Histopaque 1077 (Sigma Aldrich) gradient. The cells (1×107) were cryopreserved in 90% Fetal Bovine Serum (Cultilab) and 10% Dimethyl Sulfoxide S at -196 ºC until use. The experimental design was approved by the Ethics Committee on the Use of Animals of IVB (protocol number 003/2020) and the Ethics Committee of the Federal University of Minas Gerais (CEUA-UFMG) under protocol number 190/2018.

#### Amplification of the IGHV horse repertoire

Total RNA was extracted (Fig. 5) using the TRIzol method (Rio et al. 2010), and RNA concentrations were verified using the Qubit RNA BR Assay kit (Thermo Fisher Scientific). Following the manufacturer’s instructions, approximately 500 ng of RNA was used for cDNA synthesis using SuperScript IV enzyme (Thermo Fisher Scientific). Amplification of IGH from IGHV gene segments and the constant region was performed using multiplex PCR. A set of specific forward (F) primers for the variable region of the heavy chain (Manso et al. 2019) was used, along with specific reverse (R) primers for the constant region of the heavy chain designed previously by Navas et al. (2022). The coverage and design of the F primers was evaluated in the work of Manso et al. (2019), amplifying all known genes. The PCR parameters were as described by Navas et al. (2022).

#### Library preparation and horse IGHV repertoire sequencing

Purified DNA samples were quantified using a Qubit DNA High Sensitivity Kit (Thermo Fisher Scientific). Each amplicon was then used to prepare sequencing libraries using the Nextera XT DNA Library Prep kit (Illumina) according to the manufacturer’s instructions. For indexing PCR, the Platinum Taq DNA Polymerase High Fidelity Kit (Thermo Fisher Scientific) was used. Each horse and isotype (IgG) sample was labeled with a distinct combinatorial dual-index combination during PCR, as described by Navas et al. (2022).

After library preparation and quantification, sequencing of horse antibody heavy chains was performed. This procedure was carried out on an Illumina MiSeq 2 × 300 bp platform (Fig. 5). The different samples from each horse were pooled equally so that, when combined, they reached a final concentration of 18 pM. These solutions also received a sufficient volume of the PhiX Sequencing Control v3 (Illumina) to a final concentration of 5%. Sequencing was performed using a MiSeq System (Illumina Instrument No. M02832) at the ICB-UFMG Multiuser Laboratory Center with 301 sequencing cycles for each read, forward, and reverse sequence.

### Pre-processing and annotation of the horse IGHV repertoire

The following pre-processing steps were done equally for the eight samples. Raw Illumina MiSeq reads were pre-processed using the Immcantation framework (Gabernet et al. 2024). A fasta file containing unique sequences was obtained for IgG. Reads quality were filtered using a Phred score of > = 30 to the overlapping region, while a threshold of > = 20 was used for the read ends, using a python script available at https://github.com/JulianaEdelvacy/Gene-Conversion-Analysis represented in Fig. 5. After filtering, Ig genes were annotated using IMGT/HighV-QUEST (Alamyar et al. 2012), and the unique sequences were obtained using a script in R, available at https://github.com/JulianaEdelvacy/Gene-Conversion-Analysis, unique annotated productive sequences were used in subsequent steps.

### Modifications to BrepConvert for identifying gene conversion events in equine immunoglobulins

After annotation, the in-frame nucleotide sequences with IMGT gaps were submitted to BrepConvert (Mallaby et al. 2023) with modifications (Fig. 5). In this study, 29 in-frame functional genes (Supplementary Table 2) with their alleles, and 23 in-frame pseudogenes (Supplementary Table 3) were analyzed. Because the original program exhibited prolonged runtimes when processing Illumina sequencing data, we optimized BrepConvert to enhance computational efficiency. The first modification implemented pBlat (parallelized BLAT) to accelerate sequence alignment (Wang and Kong, 2019). Further improvements were introduced to refine the alignment of functional genes with the repertoire, by making a single alignment of all functional genes to the repertoire genes using the Biostrings package in RStudio. All modifications and source code are publicly available on the project’s GitHub repository:https://github.com/Fraternalilab/BrepConvert/. For performance assessment, 1,000 randomly selected sequences from the repertoires of eight horses were analyzed. The execution times of both the original and optimized versions were recorded, and the procedure was repeated 30 times to enable statistical comparison. All statistical analyses were performed using GraphPad Prism (version 9.1.0; GraphPad Software, San Diego, CA, USA). Data distribution was assessed using the D’Agostino–Pearson, Anderson–Darling, Shapiro–Wilk, and Kolmogorov–Smirnov normality tests. Differences between two independent groups (Normal vs. Modified) were evaluated using the Mann–Whitney test using an p < 0.0001.

#### Improving gene conversion detection fidelity using gene order filter

Gene conversion events occur only when a pseudogene is located 5’ of the target functional gene (Reynaud et al. 1987; Thompson and Neiman 1987; McCormack and Thompson 1990), making it crucial to exclude events in which the identified pseudogene is positioned 3’ of the functional gene. Therefore, we created a table with gene positions in the equine IGH locus (Equus caballus) based on the IMGT reference table (IMGT Repertoire (IG and TR). An R script was developed to filter the genes based on their genomic order (Fig. 5). If the identified pseudogene was located 3’ of the target functional gene, it was excluded; otherwise, it was retained for further analysis. This update was implemented in the BrepConvert software for all species available in IMGT, automatically importing data from the available IMGT tables and filtering the results obtained from BrepConvert. All the modifications of BrepConvert are available on the link: https://github.com/JulianaEdelvacy/Gene-Conversion-Analysis.git and on the software page https://github.com/Fraternalilab/BrepConvert.

### Evaluation of gene conversion event characteristics in horse IGHV

#### Calculation of gene conversion event percentage in equine IGHV

The frequency of gene conversion events in the IGHV region was analyzed using a Python script that employed the pandas library (McKinney 2011) for data manipulation and NumPy (Harris et al. 2020) for numerical computations on the filtered BrepConvert output. The program identifies minimum and maximum values of the start and end positions of identified gene conversion events, and defines the analysis range. A function then calculates how frequently each position within this range belongs to a gene conversion event by iterating through each position and counting its inclusion in the recorded events. With the processed data, the code calculates gene conversion event percentage for each analyzed position. The results were stored in a new DataFrame and exported to a CSV file for subsequent analysis.

#### Evaluation of pseudogene and functional gene contributions to horse IGHV gene conversion

To analyze the relationships between pseudogenes and functional genes involved in the generation of antibody diversity, we used a chord diagram. For this purpose, we employed R circlize library (Gu et al. 2014) to create the chord diagram and dplyr (Wickham et al. 2025) for data manipulation. We used the BrepConvert output containing pseudogenes and functional gene names that contributed to gene conversion events. A frequency table was created to quantify the pseudogene-functional gene combinations.

#### Analysis of 5’ and 3’ conservation between functional and pseudogenes and leader regions

Conservation data for the 5′ region between functional and pseudogenes were calculated in RStudio (version 4.5.1) using the “fiveprime identical length” and the “threeprime identical length” parameter from the BrepConvert output table. Leader regions were isolated from IMGT gene Databank, selecting the parameter “L-INTRON-L”. Alignments were made using Jalview version 2.11.5.1.

#### Identification of non-B DNA motifs in gene conversion events

Non-B DNA motifs within V-region sequences involved in gene conversion events were identified using the Non-B DNA Motif Search Tool (Cer et al. 2012a,b). The analysis was conducted on a set of 35,216 sequences associated with gene conversion events obtained in Topic 3.4.1. The motifs identified by the software were located within the sequences containing gene conversion events using an R script available on GitHub. These results were then compared with an equal number of randomly selected V-region sequences from the same horses with no detectable gene conversion. Data processing and statistical analyses were performed in RStudio and GraphPad Prism (version 9.1.0).

#### Association of gene conversion positions with AID hotspots

The overlap between AID hotspot motifs and gene conversion events was evaluated using a custom R script. Coordinates of gene conversion events were obtained as detailed in the section: Calculation of Gene Conversion event percentage in equine IGHV. Positions of AID hotspot motifs were extracted from the IMGT High-V-QUEST annotations of immunoglobulin repertoires from eight horses, utilizing the V-REGION-MUTATION-HOTSPOTS output files. Only productive sequences were included in the analysis. The AID hotspot motifs examined comprised: (a/t)(a/g)c(c/t) (WRCY; column X.a.g.g.c.t..a.t.), (a/g)g(c/t)(a/t) (RGYW; column X.a.t..a.g.c.c.t.), t(a/t) (TW; column t.a.t.), and (a/t)a (WA; column X.a.t.a.). For each gene conversion event, defined by its annotated start and end positions, overlap with AID hotspot positions was calculated using the R script. This approach leveraged IMGT-annotated files, providing a unified coordinate system for both mutation hotspots and gene conversion events. Overlaps were categorized as full overlap when the gene conversion event was entirely contained within an AID hotspot motif, partial overlap when only a segment of the event overlapped a hotspot, or no overlap when no intersection was detected. Quantification of these categories facilitated the assessment of colocalization between predicted gene conversion events and AID hotspot motifs.

#### Identification of gene conversion event motifs using MEME platform

Potential sequence motifs associated with gene conversion events were identified using the MEME platform (Bailey et al. 2015). Motif discovery was conducted with STREME (Bailey, 2021) using 10-nucleotide regions immediately upstream (5’) and downstream (3′) of each repertoire sequence with identified gene conversion events as input. The resulting motifs were compared to known regulatory motifs from databases such as JASPAR (Castro-Mondragon et al. 2022) using Tomtom (Gupta et al. 2007) with default settings. All analyses were performed independently for each horse, and statistical comparisons were carried out using Kruskal-Wallis test, followed by Dunn’s multiple comparisons test to assess specific differences, particularly comparing the reference group against all others. Two-tailed P values of ≤ 0.05 were considered statistically significant.

### Discussion

Identification of gene conversion events is essential for understanding genetic diversity, genome evolution, and the molecular mechanisms underlying diseases and immune responses. In immunoglobulins, gene conversion elucidates how species such as chickens and rabbits generate a diverse antibody repertoire to combat rapidly evolving pathogens (Becker and Knight 1990; Seo et al. 2024). Previous studies have shown that the equine immunoglobulin repertoire is characterized by limited V(D)J gene segment diversity, as demonstrated using different methodological approaches, including 5′ RACE and multiplex PCR with distinct primer sets (Tallmadge et al. 2013; Manso et al. 2019; Wibmer and Mashilo 2022; Navas et al. 2022). This restricted combinatorial diversity may constrain diversification of the antibody repertoire. In this context, we investigated whether gene conversion contributes to antibody diversity in horses. This was achieved through systematic identification of gene conversion events using an optimized high-throughput BrepConvert pipeline (Mallaby et al. 2023), a tool designed to detect gene conversion in immunoglobulin sequences with low false-positive rates.

We analyzed 754,233 annotated equine IGHV sequences, representing a more than fivefold increase compared to prior chicken immunoglobulin studies (Mallaby et al. 2023). This expanded dataset enhances detection resolution, reinforcing conclusions regarding gene conversion’s role in equine antibody diversification. Gene conversion events were identified as mismatches between functional and repertoire genes that are similar to pseudogenes. We assessed the presence of these events in approximately 6.9% of sequences per horse, a frequency comparable to the 6.61% reported for human IGHV3-23*01 (Duvvuri and Wu 2012) and substantially higher than the 0.5–0.8% estimated for mouse IgM heavy chains (Baker and Read 1995). Although methodological differences limit direct comparisons, these findings collectively suggest that gene conversion broadly contributes to heavy-chain diversification across mammals.

Our data revealed that regions associated with gene conversion in equine IGHV were predominantly short, with most detected mismatch events spanning three nucleotides, while long events (> 100 nt) accounting for only ~ 1% of cases. However, these short events likely represent the minimal detectable footprint of gene conversion events rather than their full biological extent. In other species, minimum reported event lengths vary widely, ranging from 2 to 3 nucleotides in humans and mice to 8–12 nucleotides in rabbits and chickens (Reynaud et al. 1989; Becker and Knight 1990; McCormack and Thompson 1990; Baker and Read 1995; Duvvuri and Wu 2012). However, the scarcity of systematic cross-species analyses and heterogeneity in experimental designs and analytical methods complicate direct comparisons.

Given the predominance of short mismatch events in equine IGHV, we evaluated whether these events could represent somatic hypermutation (SHM) rather than gene conversion. SHM typically involves single base pair substitutions, with insertions, deletions, and clustered mutations occurring at lower frequencies (Wilson et al. 1998; Pilzecker and Jacobs 2019; Sepúlveda et al. 2022). Although both SHM and gene conversion are initiated by AID, they produce distinct mutational signatures. SHM typically introduces point mutations directly at AID target motifs and is enriched within CDR regions (Bothwell et al. 1982; Wang et al. 2023). In contrast, gene conversion can generate contiguous events of sequence replacement that extend beyond the initial AID-induced lesion (McCormack and Thompson 1990). Our analysis revealed that, in our dataset, mismatched regions are enriched adjacent to, but not within, AID-targeting motifs, which is consistent with patterns observed in chickens (Arakawa et al. 2002; Mallaby et al. 2023). Additionally, the enrichment of mismatched regions in framework regions, particularly FR1, contrasts with the typical distribution of SHM, which is more concentrated in CDRs. Together, these features support the interpretation that the identified mismatched regions are more likely associated with gene conversion events.

Structurally, gene conversion in horses exhibits distinct features. Events are strongly enriched in FR1, contrasting with chickens, where gene conversion is CDR3-biased (Reynaud et al. 1987), and rabbits, where it is distributed across framework regions and CDRs (Becker and Knight 1990). This may reflect species-specific diversification organization or methodological differences, as recent studies identified gene conversion across various V gene regions in chicken immunoglobulin (Mallaby et al. 2023). In horses, gene conversion preferentially involves IGHV4 family genes, the most abundant functional IGHV family, paralleling biases toward IGHV1 in rabbits and IGHV3-23*01 in humans (Becker and Knight 1990; Duvvuri and Wu 2012). The inversely oriented IGHV4-21 gene exhibited the highest conversion frequency, consistent with orientation-dependent recombination models (McCormack and Thompson 1990).

Analysis of sequence identity between frequently used functional genes and pseudogenes involved in gene conversion revealed similarities below 80%, lower than minimum values reported previously (McCormack and Thompson 1990). However, prior research emphasizes that while high identity between pseudogenes and functional genes is common in gene conversion, the critical determinant is the presence of identical flanking regions, particularly in the 5’ region of the event. This suggests that high overall sequence identity alone does not fully explain gene conversion occurrence, underscoring the importance of local sequence identity in flanking regions. Our investigation showed that most gene conversion events exhibited identical 5’ flanking regions of six nucleotides, and one nucleotide at 3’, consistent with findings by Reynaud et al. (1987) and McCormack and Thompson (1990), supporting the role of local sequence identity in facilitating gene conversion even in low-identity sequences.

Specifically, 91% of the detected 3-nucleotide mismatch events were flanked by these conserved regions, suggesting that the underlying gene conversion events extend beyond the mismatched segment, reaching at least ~ 10 nucleotides. Additional sequence identity observed in leader regions between functional genes and pseudogenes further supports the presence of extended conversion events, potentially ranging from 15 to 36 nucleotides. Thus, the 3-nucleotide segments identified here should be interpreted as minimal detectable units within larger gene conversion events, consistent with previously reported events lengths. Furthermore, previous studies indicate that short conversion events promote sequence diversification rather than homogenization (Takuno et al. 2008). This pattern is particularly evident at loci under diversifying selection, such as MHC/HLA (Widera and Flavell 1984; Jeffreys and May 2004; Miller and Lambert 2004; Khan et al. 2022), where diversity is critical for effective immune responses. Mechanistically, this aligns with polymerase η–mediated repair, favoring synthesis events during homologous recombination (Kawamoto et al. 2005; Chakraborty et al. 2023).

Finally, the association of gene conversion events with direct repeat motifs and a conserved downstream ZN691 binding site suggests cooperation between DNA structural features and homologous recombination repair pathways in equine gene conversion (Chuzhanova et al. 2009; Hwang et al. 2019). The high prevalence of direct repeats and slipped motifs corroborates prior findings (Chuzhanova et al. 2009) indicating these motifs’ involvement in gene conversion. Those results provide evidence that non-B DNA conformations may contribute to gene conversion by inducing DNA double-strand breaks that activate recombination machinery (Supplementary Fig. 6). The presence of a binding site for the C2H2 zinc finger family member ZN691 implies participation in DNA double-strand break repair, supported by previous studies (Hwang et al. 2019; Pieraccioli et al. 2016; Kamaliyan and Clarke 2024; Singh and van Attikum 2021).

## Conclusion

Despite the limited usage of IGHV segments, our findings suggest that horses exploit pseudogenes to diversify their primary IGHV repertoire through gene conversion. The preferential localization of these events within FR1—a region generally disfavored for somatic hypermutation, together with their high sequence identity to pseudogenes and occurrence outside AID hotspots, supports a distinct diversification mechanism. To our knowledge, this is the first study to characterize gene conversion in equine IGHV genes. The biased usage of specific pseudogenes (IGHV4-35, IGHV4-53, IGHV4-38) and functional genes (IGHV4-21, IGHV4-22, IGHV4-29), combined with the observation that short mismatch events likely represent longer events (15–36 nucleotides), further highlights the structured nature of this process. Moreover, the association with non-B DNA conformations and ZNF691 suggests a potential influence of local DNA structure and DNA-binding factors. Collectively, these findings establish gene conversion as a relevant and potentially regulated mechanism of antibody diversification in horses, providing new insights into species-specific immune strategies and a foundation for future studies of equine immune responses.

## Supplementary Information

Below is the link to the electronic supplementary material.

Supplementary Material 1 (DOCX 662 KB)

## Data Availability

Four of the sequences used in this study were previously deposited in the NIH Sequence Read Archive (SRA) under accession number PRJNA851406 for the work of Navas et al. (2022). The remaining four horse samples generated for this study have been deposited in the NIH SRA under accession number PRJNA1345687.

## Code availability

The updated computational pipeline described above, which is used to annotate gene conversion events, is available as the version 0.2.0 of the R package ‘BrepConvert’ at https://github.com/Fraternalilab/BrepConvert. The code used for subsequent analysis of annotated gene conversion is available at https://github.com/JulianaEdelvacy/Gene-Conversion-Analysis.git.
