# Supplementary material for: Identification of gene conversion events in horse IGHV suggests preferential hotspots for diversification
Source: Immunogenetics. 2026 May 19;78(1):8. doi: 10.1007/s00251-026-01400-7 (PMC13183705; doi:10.1007/s00251-026-01400-7)
Supplement: Supplementary file 1 — Supplementary Material 1 (DOCX 662 KB) [file 251_2026_1400_MOESM1_ESM.docx]

**Supplementary information for:**

**Identification of Gene Conversion Events in Horse IGHV Suggests Preferential Hotspots for Diversification**


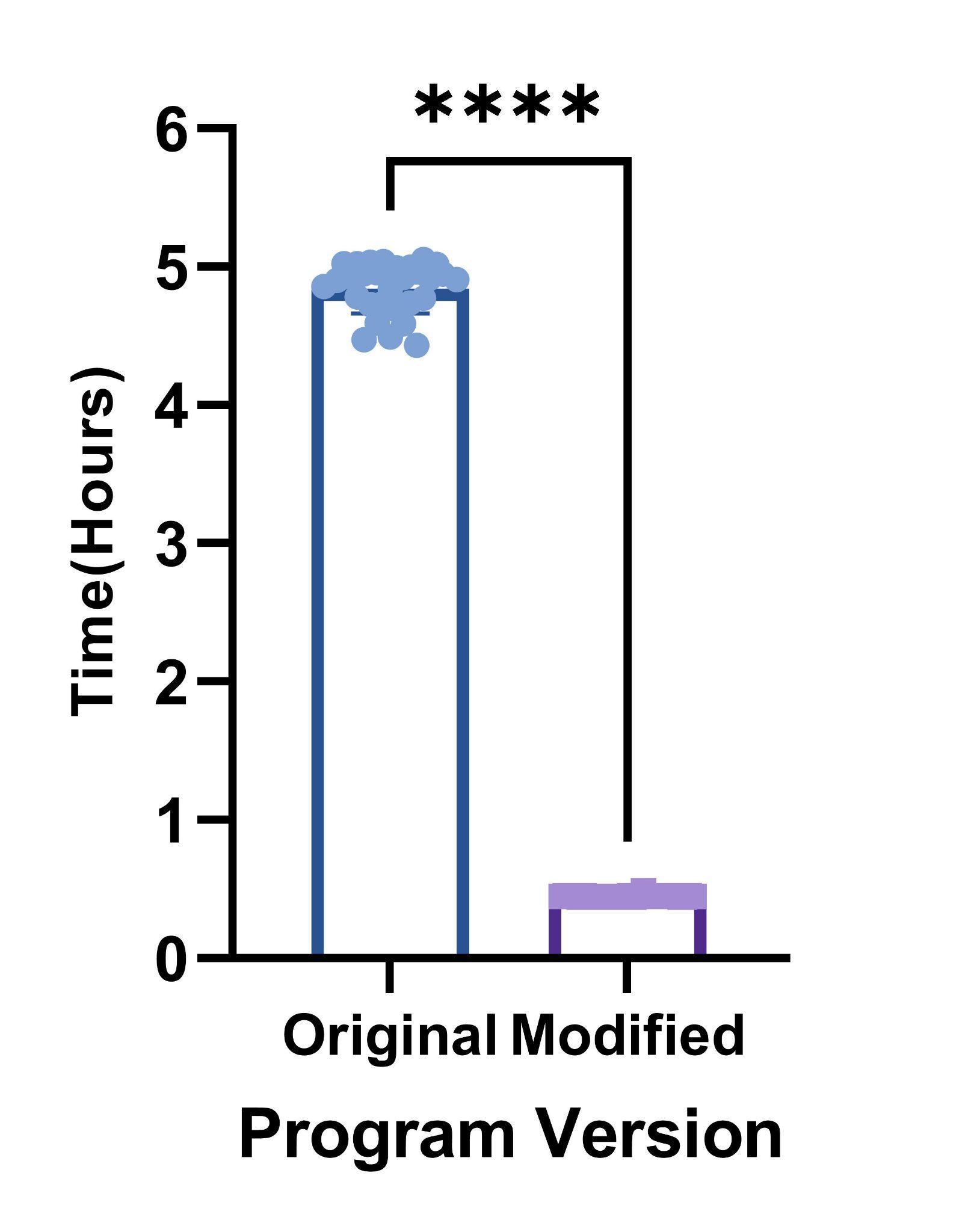


**Supplementary Figure 1:** BrepConvert processing time before and after implementation of pBLAT for gene conversion identification. Execution time was measured by randomly selecting 1,000 sequences from the annotated antibody repertoire of eight horses and repeating the process 30 times using either the original software (blue) or the optimized version (purple). Statistical significance was assessed using the Mann–Whitney test. ****p < 0.0001.

**
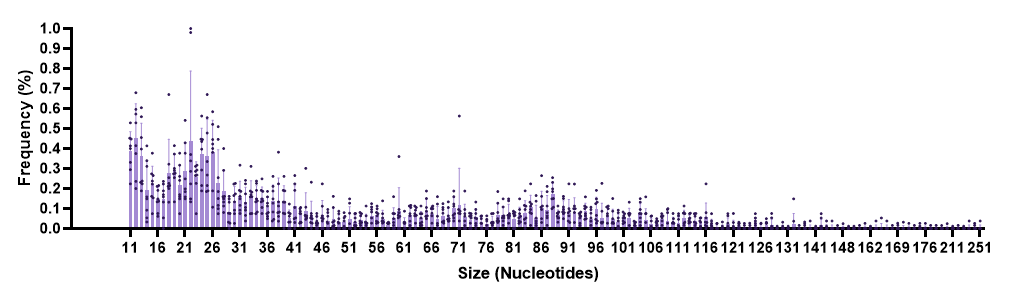
**

**Supplementary Figure 2:** Percentage of the size in nucleotides of gene conversion events in horse IGHV higher than 10 nucleotides.


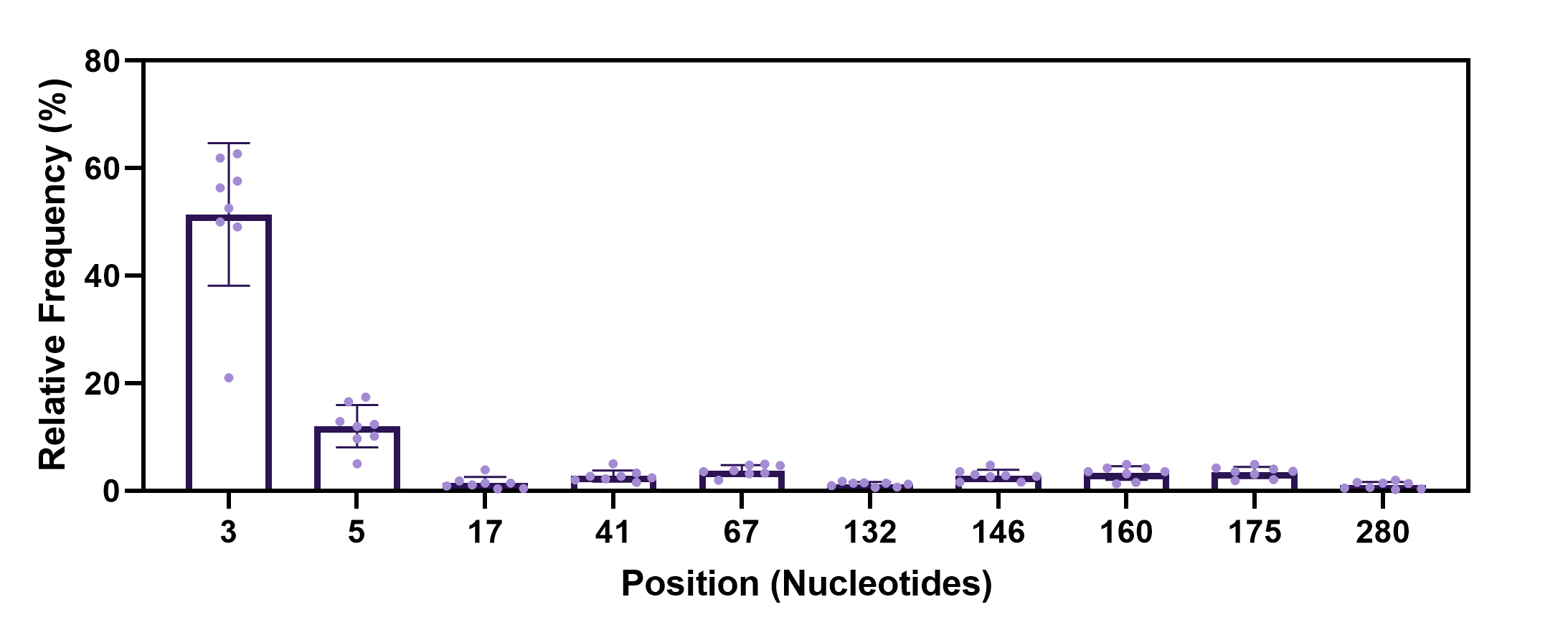


**Supplementary Figure 3:** Nearest Gene ConverAID Motif Position. This figure shows the position of the nearest AID motif from the observed gene conversion events. The highest observed values correspond to positions with the greatest number of gene conversion events in our dataset.

**Supplementary Table 1**: Summary of Motif and Event Distances

| **Description** | **Range** |
| --- | --- |
| Distance to AID Motif | 0-35 |
| Position of AID Motif | 3-310 |
| Size of gene conversion Event | 3-251 |
| Distance between Repertoire and Pseudogene | 0-49 |

Source: This table summarizes the distances and sizes related to the AID motif and gene conversion events


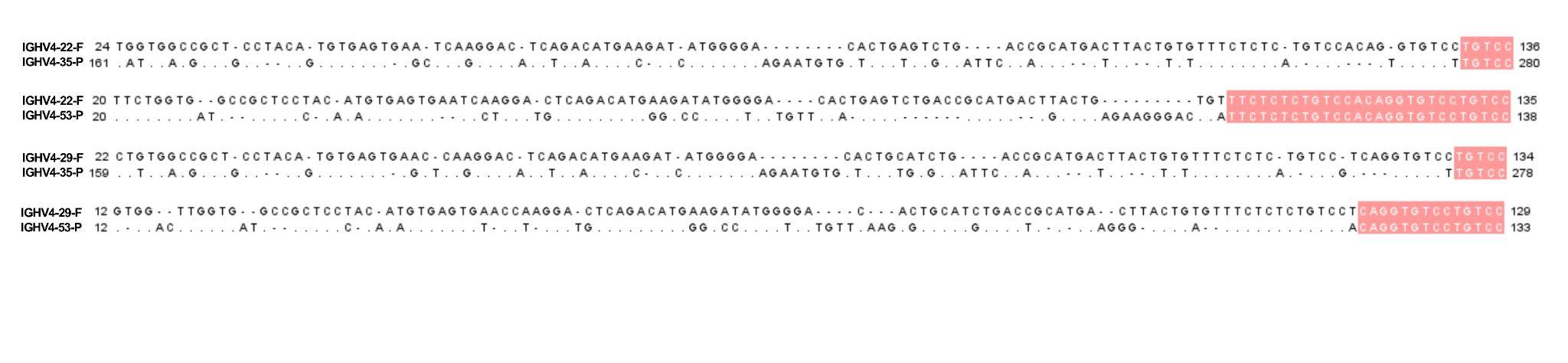


**Supplementary Figure 4:** Alignment of Leader regions between functional and pseudogenes most frequent in IGHV gene conversion events. Identical regions are highlighted in light red. Dots indicate positions that are identical to the functional sequence. Alignments were made using Jalview version 2.11.5.1.


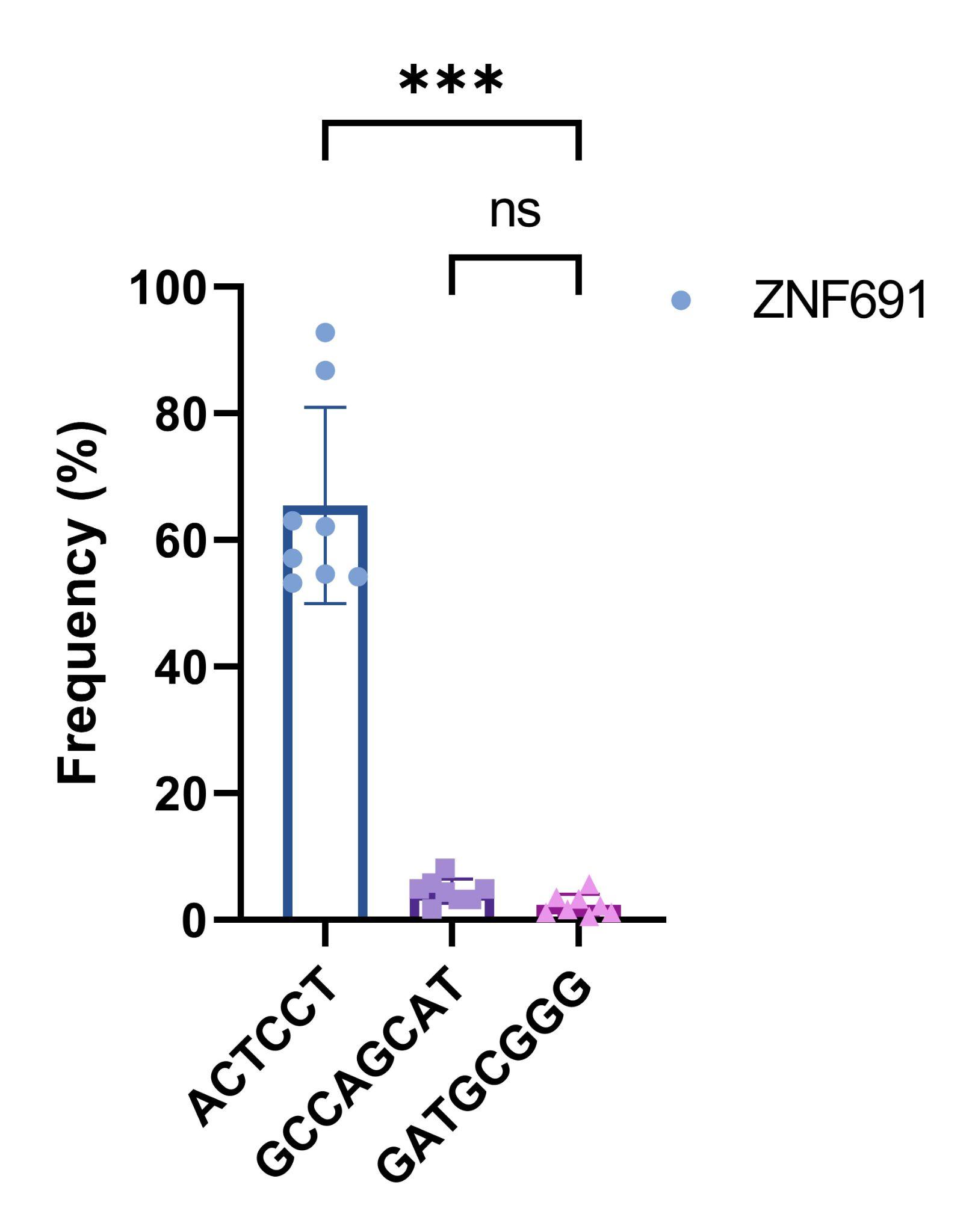


**Supplementary Figure 5:** Identification of motifs downstream of expanded gene conversion events. Motifs identified within a 10-nucleotide region downstream (3′) of the expanded gene conversion sites. Statistical significance was assessed using the Kruskal–Wallis test. Significance was determined using a Kruskal-Wallis test. ***p = 0.0001. ns p = 0.5365. Sequences representative of the identified motifs.

**Supplementary Table 2**: Equine Functional IGHV Gene Sequences

| **Sequence ID** | **Gene Name** | **Nucleotide Sequence** |
| --- | --- | --- |
| NW 001876796 | IGHV1-41*01 | gaggtccagctggtgcagtctggggct...gaggtgaggaagccaggggcatccgtgaag gtctcctgcaaggcttctggagacagcttc............acttattactctatgagc tgggtgcgacaggcccctggacaagggctcgactggatgggagggatcttgcctata... ...gttgatgatacaagctacacgcagaagttccag...ggcagagtcaccatgactgca gacaag...tccacgagcacagtctacatggagctgagcagtctgacatcc gaggacacggccgtgtattactgtgcaaaaga |
| IMGT000040 | IGHV1-41*02 | ................................................................................................... .................................................................................... gttgatgat-  acaagctacacgcagaagttccag...ggcagagtcaccatgactgcagacaag...tcca cgagcacagtctacatggagctgagcagtctgacatccgaggacacggccgtgtattactgtgcaacaga |
| IMGT000040 | IGHV1-41D*01 | ................................................................................................  .......................................................................................gttgat-  gatacaagcta cacgcagaagttccag...ggcagagtcaccatgactgcagacaag...tc cacgagcacagtctacatggagctgagcagtctgacatccgaggacacggccgtgt attactgtgcaacaga |
| IMGT000040 | IGHV1-41N*01 | ..............................................................................................  .........................................................................................gttgat-  gatacaagctacacgcaga agttccag...ggcagagtcaccatgactgcagacaag...tcc acgagcacagtctacatggagctgagcagtctgacatccgaggacacg gccgtgtattactgtgcaacaga |
| IMGT000040 | IGHV1-5*01 | gaggtccagctggtgcagtctggggct...gaggtgaagaagccaggggcatccgtgaag gtctcctgcaaggcttctggagacagcttc............acttattactctatgagc tgggtgcgacaggcccctggacaagggcttgagtggacgggatatatctatcctgaa... ...tatgatgctatgggctacccgcagaagttccag...ggcagagtcaccatgactgcg gacaag...tccacgagcacagtctacatggagctgagcagtctggcatctgaggacaca gccgtgtattactgtgcaacaga |
| IMGT000040 | IGHV1-70*01 | gaggtccagctaatacagtcggggcca...gagttgaagaagcctgggtcatcagtgaag atctcctgcaaggcttctggatacaccttc............actgaatatgctatgcac tgggtgcgacaggccaatggaaaagggattgaatggatgggatctatcagtcctcat... ...gatgatgatacgagctacgcaccgaagttccaa...ggcagagtcaccatcaccgtg gacaag...tccacgagcacagtctacatggagctgagcagtctgacatctgaggacacg gccatgtattactgtgcgaaaga |
| NW 001876796 | IGHV1-70*02 | gagggtcagctggaacagtcggggccg...gagttgaagaagcctgggtcatcagtgaag atctcctgcaaggcttctggatacaccttc............agtagctatgctgtgcac tgggtgcgacaggccaatggaaaagggattgagtggatgggatctatctatgctgaa... ...tatgatgatacaagctacgcaccgaagttccag...ggcagagtcaccatgactgcg gacaag...tccacgagcacagtctacatggagctgagcagtctgacatctgaggacatg gccgtgtattactgtgcaacaga |
| NW 019646341 | IGHV1-70*03 | gagggtcagctggaacagtcggggccg...gagttgaagaagcctgggtcatcagtgaag atctcctgcaaggcttctggatacaccttc............agtagctatmctgtgcac tgggtgcgacaggccaatggaaaagggattgagtggatgggatctatctatgctgaa... ...tatgatgatacaagctacgcaccgaagttccag...ggcagagtcaccatgactgcg gacaag...tccacgagcacagtctacatggagctgagcagtctgacatctgaggacacg gccgtgtattactgtgcaacaga |
| IMGT000040 | IGHV2-63*01 | cagatcagcctgcaggagtctggtcct...gggctgctgaagcccacccagacccttacg ctgacctgctccttctctgggttctcactgact......acttctgatattggtgttggt tggatgcgtcaaccccctgggaaggcactggagtggctcacctatgtttggtggact... ......gatgaaaagcattacaacccatctctgaag...agccggctcacaatctccaag gacacc...tccaaaaaccaggtgatgctgacaatgaccagtttggaccctccagacaca gccacatattactgtgtaaagaggg |
| IMGT000040 | IGHV3-78*01 | gaggtgcagctggtggagtctggggga...ggcctggtgaagcctggggggtctctgaga ctctcctgtgcagcctctggattcaccttc............agcagctatgctatgagc tgggtccgccaggctccaggaaagggcctgcagtgggtcgcaggtattaacagtgat... ...ggtggtagcacacactacgccgactccgtgaag...ggccgattcaccatctccaga gacaac...gccaagaacacggtgtgtctgcaaatgaacagcctgagagacgaggacacg gccgtgtattactgtgcgaagga |
| IMGT000040 | IGHV4-11*01 | caggtgcaactgaaggagtcaggacct...ggcctggtgaagccctcgcagaccctgtcc ctcacctgccctgtctctagattcccttta............accaaccatcatgtacac tggacccaccaggctccaggaaaagggctggagtggcttggtgattcaaggagtggt... ......gaaagcacatactacaacttaactctgaag...tcccaactcagcatccccagt gatact...tccaaaagccaaatttatttaacgctgaacaggctgagaggcgatgacatg gccatgtactactgtgccagaga |
| IMGT000040 | IGHV4-17*01 | caggtgaagctgcaggagtcgggccca...ggactggtgaagccctcacagaccctctcc ctcacctgctctgtgtctggagtctccatcaca......agcagtggtgactggtggagc tggatccgccagcccccagggaaggggctggaatggatggggtacataagttatagt... ......ggtagcgcttactacaccacatccctcaag...agccgactctccatctccaga gacacg...tccaaggaccagttctccctgcagctgagctccgtgaccgccgaggacacg gccgtttattactgtgcaaggaag |
| NW 001876796 | IGHV4-17*02 | caggtgaagctgcaggagtcgggccca...ggactggtgaagccctcacagaccctctcc ctcacctgctctgtgtctggagtctccatcaca......agcagtggtgactggtggagc tggatccgccagcccccagggaaggggctggaatggatggggtacataagttatagt... ......ggtagcgcttactacaccacatccctcaag...agccgactctccatctccaga gacacg...tccaaggaccagttctccctgcagctgagctccgtgaccacagaggacacg gccgtttattactgtgcaagtga |
| IMGT000040 | IGHV4-21*01 | caggtgcaactgaaggagtcaggacct...ggcctggtgaagccctcgcagaccctctcc ctcacctgcactgtctctggattatctttg............agcagttatggtgtgggc tgggtccgccaggctccaggaaaagggctggaatttgttggtggtatagctagtagt... ......ggaagtgcaaactacaacccagccctgaag...tcccgagccagcatcaccaag gacacc...tcaaagagccaagtttatctgacgctgaacagcctgacaagcgaggacacg gccgtctattactgtgcaggagg |
| IMGT000040 | IGHV4-22*01 | caggtgcaactgaaggagtcaggacct...ggcctggtgaagccctcgcagaccctgtcc ctcacctgcactgtctctggattctctttg............agcagttacggtgtaggc tgggtctgccaggctccaggaaaagggctggaatatgttggtgttatagctagtagt... ......ggaagtgcaaactacaacccagccctgaag...tcccgagccagcatcaccaag gacacc...tcaaagagccaagtttatctgacgctgaacagcctgacaggcgaggacacg gccgtctattactgtgcgaga |
| IMGT000040 | IGHV4-29*01 | caggtgcaactgaaggagtcaggacct...ggcctggtgaagccctcgcagaccctctcc ctcacctgcactgtctctggattatctttg............agcagttatgctgtaggc tgggtccmccaggctccaggaaaagggctggaatatgttggtgctatatatggtagt... ......gcaagtgcaaactacaacccagccctgaag...tcccgagccagcatcaccaag gacacc...tcaaagagccaagtttatctgacgctgaacagcctgacaggcgaggacacg gccgtctattactgtgcgaga |
| NW 001876796 | IGHV4-29*02 | caggtgcaactgaaggagtcaggacct...ggcctggtgaagccctcgcagaccctgtcc ctcacctgcactgtctctggattatctttg............agcagttatgctgtaggc tgggtccgccaggctccaggaaaagggctggaatatgttggtgctatatatggtagt... ......gcaagtgcaaactacaacccagccctgaag...tcccgagccagcatcaccaag gacacc...tcaaagagccaagtttatctgacgctgaacagcctgacaggcgaggacacg gccgtctattactgtgcgaga |
| IMGT000040 | IGHV4-37*01 | caggtgcagctgaaggagtcgggccca...ggacaggtgaagccctcacagaccctctcc ctcacctgcactgtcactggaggctccatcaca......agcaggtattatggctggagc tggatccgccagaccccagggaaggggctggagtacattgggagcatagcttatagt... ......ggtagcacttactacagcccatccttcaag...agccgcgcctccatctccaga gacacg...tccaagaaccagttctccctgcagctgagctccgtgaccaccgaggacacg gccgtttattactgtgcaagtga |
| NW 001872990 | IGHV4-37*02 | caggtgcagctgaagaagtcgggccca...ggacaggtgaagccctcacagaccctctcc ctcacctgcactgtcactggaggctccatcaca......agcaggtattatggctggagc tggatccgccagaccccagggaaggggctggagtacattgggagcatagcttatagt... ......ggtagcacttactacagcccatccttcaag...agccgcgcctccatctccaga gacacg...tccaagaaccagttctccctgcagctgagctccgtgaccacagaggacacg gccgtttattactgtgcaagtga |
| IMGT000040 | IGHV4-37D*01 | caggtgcagctgaagaagtcgggccca...ggacaggtgaagccctcacagaccctctcc ctcacctgcactgtcactggaggctccatcaca......agcaggtattatggctggagc tggatccgccagaccccagggaaggggctggagtacattgggagcatagcttatagt... ......ggtagcacttactacagcccatccttcaag...agccgcgcctccatctccaga gacacg...tccaagaaccagttctccctgcagctgagctccgtgaccaccgaggacacg gccgtttattactgtgcaagtga |
| IMGT000040 | IGHV4-37N*01 | caggtgcagctgaaggagtcgggccca...ggacaggtgaagccctcacagaccctctcc ctcacctgcactgtcactggaggctccatcaca......agcaggtattatggctggagc tggatccgccagaccccagggaaggggctggagtacattgggagcatcacttatagt... ......ggtagcacttactacagcccatccttcaag...agccgcgcctccatctccaga gacacg...tccaagaaccagttctccctgcagctgagctccgtgaccacagaggacacg gccgtttattactgtgcaagtga |
| IMGT000040 | IGHV4-55*01 | caggtgcagctgcaggagtcgggccca...ggactggtgaagccctcacagaccctctcc ctcacctgcactgtcactggttactccatcacc.........agtggttactactggagc tggatccgtcagcccccaggaaagaggctggagtggatgggctccatatattatagt... ......ggtagcacttactacagcccatccctcaag...agccgcatcaccatctccaca gacacg...tcccagaaccagtcctccctgcagctgagctccgtgaccaccaaggacaca gctgtgtattactgtgccagaga |
| IMGT000040 | IGHV4-59*01 | caggtgcagctgcaggagtcaggacca...ggccagacgaatccctcacagaccctgtcc ctcacatgcactgtcactggttactccatcacc.........agtggttatggctggaac tggatccgccagccaccaaacaaagggctggagtggatggggagcataagctatagt... ......ggtagaactaactacagcccatccctcagg...agccgcatcaccatctccaga gacact...tccaagaaccagttcttgctgcagctgagctcagtaaccactgaggacacg gccgtgtattactgtgcgacaga |
| IMGT000040 | IGHV4-65*01 | caggtgcagctgcaggagtcgggccca...ggactggtgcagccctcacagaccctgtcc ctcacctgcactgtcactggaggctccatcaca......agcagctattctagctggagc tggttacgccagcctccagggaaggggctggagtacatggggtacatatattatgat... ......ggtagaacttactacaatccttccttcaag...agccgcacctccatctccaga gacacc...tccaagaaccagttctccctgcagctgagctccgtgaccaccgaggacgcg gccgtgtattactgtgcaagaga |
| NW 001876796 | IGHV4-65*02 | caggtgcagctgcaggagtcggmccca...ggactggtgcagccctcacagaccctgtcc ctcacctgcactgtcactggaggctccatcaca......agcagctattctagctggagc tggttacgccagcctccagggaaggggctggagtacatggggtacatatattatgat... ......ggtagaacttactacaatccttccttcaag...agccgcacctccatctccaga gacacc...tccaggaaccagttctccctgcagctgagctccgtgaccaccgaggacgcg gccgtgtattactgtgcaagaga |
| IMGT000040 | IGHV4-75*01 | caggtgaagctgcagagttggggccca...gaacttgtgaagccctcgcagacaatctcc ctcacttgtgctgtctatggatcctccttcaca......agcagtgattatggttggagc tggactcgccagcccctaaggaaggggctggagtggatgggacttgtaagctatagt... ......ggtagcacttactacagcccatccctcaag...agccgcatctccatctccaga gacacg...tccaagaaccagttctccctgcagctgagctccgtgaccaccgaggacacg gccgtgtattactgtgcaagagtgaaagttagatattatgatgca |
| IMGT000040 | IGHV4-82*01 | caggtgcaactgaaggagtcaggacct...ggcctggtgaagccctcgcagaccctctcc ctcacctgcactgtctctggattctctttg............agcagttatgctgtaggc tgggtccgccaggctccaggaaaagggctggaatatgttggtgctatatatggtagt... ......gcaagtgcaaactacaacccagccctgaag...tcccgagccagcatcaccaag gacacc...tccaagagccaagtttatctgacgctgaacagcctgacaggcgaggacacg gccgtctattactgtgcgaaaga |
| NW 001876796 | IGHV4S1*01 | caggtgcaactgaaggagtcgggacct...ggcctggtgaagccctcgcagaccctgtcc ctcacctgcactgtctctggattatctttg............agcagtaatgctgtaggc tgggtccgccaggctccaggaaaagggctggagtgggttggtgttatatatggtagt... ......gaaagtacatactacaacccagccctgaag...tcccgagccagcatcaccaag gacacc...tcaaagagccaagtttatctgacgctgaacagcctgacaggcgaagacacg gccgtctattactgtgcaggatg |
| IMGT000040 | IGHV9-66*01 | gaggaccctctcgtgcaatggggaggt...ggagtggtggtctcctcacagacactcagc ctcacctgtgccgcctacaaacgcaaagtt............tcagaatattccctgtgg tggattcgccttctcccagggaaggggttggagtgcgtaggtgtgatctgggctaag... ......ggggacactcagtgcagcccccacctgcag...tctcgagtcagcatctccagg gacgcc...accaagaaccaagtgttcttacagctgagcagtgtgatgcctgaggattca ggcgtgtattactgtgctcaaga |

**Supplementary Table 3**: Equine IGHV Pseudogene Sequences

| **Sequence ID** | **Gene Name** | **Nucleotide Sequence** |
| --- | --- | --- |
| IMGT000040 | IGHV3-58*01 | gaggtgcagctggtggagtctggggga............................................. gcctctggattcaactcc............agtatctccttggtgtactggatgcaccagg atgcagggaagggactgcgctgggttggatgaattaataacggt......ggaagtagcact aactgtgctggctctgtggag...ggctggttcaccatctcgagagacaac...agcaaga acaccctatttctccaaatgagcaacctgagagccaaggacacggctgtatattgct gtgcaggaga |
| IMGT000040 | IGHV3-69*01 | gaggtgcagctggtggagtccggggga...ggcttggtgcaacctgggtggtctctgaga gtctcatgtgcagcctctggattcatctgt............agcagctactggatggac tgggtctgccaggttccagggaaggtgctggactgggttggttgaattaatacagat... ...ggatgcagcatttactatgctgactttgtgaag...tgctgattcaccatctccaga gacaat...gacaagaacatgctgtatttgtaaatgaacagactgagcaccaagggtatg gctgtgtattaccgtgcaagacg |
| NW 001876796 | IGHV3-69*02 | gaggtgcagctggtggagtccggggga...ggcttggtgcaacctgggtggtctctgaga gtctcatgtgcagcctctggattcatctgt............agcagctactggatggac tgggtctgccaggttccagggaaggtgctggactgggttggttgaattagtacagat... ...ggatgcagcatttactatgctgactttgtgaag...tgctgattcaccatctccaga gacaat...gacaagaacatgctgtatttgtaaatgaacagcctgagcaccaagggtatg gctgtgtattaccgtgcaagaca |
| IMGT000040 | IGHV3-9*01 | gaggtgcagctggtggagtctggggga...ggcctggtgaagcctggggggtctctgaga ctctcctgtgcagcctctggattcactttt............gatgactatgccctgcac tgggttagacaggctccaggaaaaggacttgagtgggtgtcattcattagctggagt... ...ggtggtagcacacactacgctgactccgtgaag...ggccgattcaccatctccaga gacaac...gccaagaacacgctctatctgcaaatgaacagcctgagagacgaggacacg gccgtgtattattgtgcaaaaga |
| IMGT000040 | IGHV4-10*01 | caggtgctggtgtaggagtcaggccca...ggacacgtgaagccctcagagacacgctcc ctctcctggtctgtctctacattctccatcaca......aactgcagttactgctgggac tgaatccaccagcccctaggaaacggactggattggatagcgtgttttggttctgta... ......ggcagcacggtatataacctgcatctccaa...agatgactctccctctccaga gacaca...tccaagaaccagttctctctgcagctgagctccgtgaaggctgagcacacg gccctgtattactgtgcaagaga |
| IMGT000040 | IGHV4-20*01 | ccaggtgtggatcaggagtctgctcca...ggactggtgcagccctcacagacacacacc ctcacttgtgctgcctctggaatctccatcaca......accaggagataatgttgggac tggaccctcatgcctccagggaaggggctggagtggatgggagatataccttatcat... ......gggaacacagactgtaaaacgtcgctaaag...agccgcacctctgtctccaga gacatg...accaagaagcagttttccctgaagctgagctccgtgaccactgaggacaaa gctgtgagttactgtgcaagatg |
| NW 001876796 | IGHV4-20*02 | ccaggtgtggatcaggagtctggtcca...ggactggtgcagccctcacagacacacacc ctcacttgtgctgcctctggaatctccatcaca......accaggagataatgttgggac tggaccctcatgcctccagggaaggggctggagtggatgggagatataccttatcat... ......gggaacacagactgtaaaacgtcgctaaag...agccgcacctctgtctccaga gacatg...accaagaagcagttttccctgaagctgagctccgtgaccactgaggacaaa gctgtgagttactgtgcaagatg |
| IMGT000040 | IGHV4-20D*01 | ccaggtgtggatcaggagtctgctcca...ggactggtgcagccctcacagacacacacc ctcacttgtgctgcctctggaatctccatcaca......accaggagataatgttgggac tggaccctcatgcctccagggaaggggctggagtggatgggagatataccttatcat... ......gggaacacagactgtaaaacgtcgctaaag...agccgcacctctgtctccaga gacatg...accaagaagcagttttccctgaagctgagctccgtgaccactgaggacaaa gctgtgagttactgtgcaagatg |
| IMGT000040 | IGHV4-20N*01 | ccaggtgtggatcaggagtctgctcca...ggactggtgcagccctcacagacacacacc ctcacttgtgctgcctctggaatctccatcaca......accaggagataatgttgggac tggaccctcatgcctccagggaaggggctggagtggatgggagatataccttatcat... ......gggaacacagactgtaaaacgtcgctaaag...agccgcacctctgtctccaga gacatg...accaagaagcagatttccctgaagctgagctccgtgaccactgaggacaaa gctgtgagttactgtgcaagatg |
| IMGT000040 | IGHV4-35*01 | cagtcaaagtgccaggagtcagcccca...gggcaggtgaagccctcagagaccacgtcc tcagcctgcattctctctggtaactttgtcacc.........agtgatgaatcctggtgc tgggtccaccagcctccagggaaggggctggaatggcttgggcatggtcactatagggca tcatcatggaacacagactacaacccatgcatcaag...gaccgcatctccatcactgct gacatcacttccaagaaatagttttccctgcagctgagctttgtggtggctgaggacaca gtgatgtatctctgcacaaga |
| NW 001876796 | IGHV4-35*02 | cagtcaaagtgtcaggagtcagcccca...gggcagatgaagccctcagagaccacgtcc tcagcctgcattctctctggtaactttgtcacc.........agtgatgaatcctggtgc tgggtccaccagcctccagggaaggggctggaatggcttgggcatggtcactatagggca tcatcatggaacacagactacaacccatgcatcaag...gaccgcatctccatcactgct gacatcacttccaagaaatagttttccctgcagctgagctttgtggtggctgaggacaca gtgatgtatctctgcacaaga |
| IMGT000040 | IGHV4-35D*01 | cagtcaaagtgccaggagtcagcccca...gggcaggtgaagccctcagagaccacgtcc tcagcctgcattctctctggtaactttgtcacc.........agtgatgaatcctggtgc tgggtccaccagcctccagggaaggggctggaatggcttgggcatggtcactatagggca tcatcatggaacacagactacaacccatgcatcaag...gaccgcatctccatcactgct gacatcacttccaagaaatagttttccctgcagctgagctttgtggtggctgaggacaca gtgatgtatctctgcacaaga |
| IMGT000040 | IGHV4-38*01 | caggtgcagctggaagagctgggccca...ggactggtgaagcccgtgatgaccatcgcc ctcacctgtgctgtctctggtttctgcatcaca......gccatctgtggctggtgcagc tgagttttccagagcccggggaaggggttggaaaggacgagatgcatctgtgatggt... ......ggtagcacaacttataacccaacacgcaaa...agctggagctctgtccccaga gacatg...tccaagaaccagttctccctgccgctgagctctctgatcaggaaggacaca gctgtgtgttactgcgcaacatg |
| IMGT000040 | IGHV4-38N*01 | caggtgcagctggaagagctgggccca...ggactggtgaagcccgtgatmaccatcgcc ctcacctgtgctgtctctggtttctgcatcaca......gccatctgtggctggtgcagc tgagttttccagagcccggggaaggggttggaaaggatgagatgcatctgtgatggt... ......ggtagcacaacttataacccaacacgcaaa...agctggagctctgtccccaga gacatg...tccaagaaccagttctccctgccgctgagctctctgatcaggaaggacaca gctgtgtgttactgcgcaacatg |
| IMGT000040 | IGHV4-52*01 | caggtgcagctgcaggagtcgggccca...ggactggtgcagccctcacagaccctgtcc ctcacctccactgtctatggattctccatcaca......accagctgatactgatggaac tggatccacgagaccccagagaaggggctgctgtggattgggcagaaatgttataat... ......gggaacactaactacagcccatccctcaaa...agctgcaactacatctgcaga gatatg...atcaagaaccagttctccctgcaattgagccatttgaccactaaagacacg atcatgtattacaggggaaggga |
| IMGT000040 | IGHV4-53*01 | caggtgaagctgcaggaataggggtca...gacctggtgaagccctcgcagaccctctcc ctcacttgtgctgtctctggattctccatcaca......acagactattactcctggatg tggatccgccagcccccaggaaaaagcctggagtacatggggcacatacattctaat... ......gggaacacagattacaaaccttcctttgag...agccacatctccatctccata gataca...tccaagaaccagttctccctgcagctgaactcgatgactgccgaggacaca gctgtgtattactgtgccaga |
| IMGT000040 | IGHV4-57*01 | caggtgcagctgcagaagttgagccca...ggattggtgaagacctcacagaccatctca ttcaactgtcctgtctatggattctccatcacc.........agtggttatgaatggatg aggatttgtcagtccccagggaagggctttgagtagatgaggtacataaatgctgct... ......ggtagcacagcatacaatccatccctcaag...agctgcatctttatctcgaga aactca...tccaagaacaagttctccctgaagctgagctccatgactgttaaggagaga gccacgtattactattcaaga |
| IMGT000040 | IGHV4-68*01 | caggtgcagctgcaagggtcattccca...ggtctagtgaagccatgacagaccatgtca ctcacctgtgctgtctgtggattccccatcatc.........agtggttatgaatgcatg tggatccagcagcccccagggcagggcttggagtggatgtggtacataaatgctgat... ......ggttgcacagcatatgacccatcccacgag...agctgcatctccatctccaga gacaag...tcccagaaacagttctccctgcagttgagctccaggaccactgtggacatg gccatctattactgtgcaagaga |
| NW 001876796 | IGHV4-68*02 | caggtgcagctgcaagggtcattccca...ggtctagtgaagccatgacagaccatgtca ctcacctgtgctgtctgtggattccccatcatc.........agtggttatgaatgcatg tggatccagcagcccccagggcagggcttggagtggatgtggtacataaatgctgat... ......ggttgcacagcatatgacccatcccgcgag...agctgcatctccatctccaga gacaag...tcccagaaacagttctccctgcagttgagctccaggaccactgtggacatg gccatctattactgtgcaagaga |
| IMGT000040 | IGHV4-72*01 | caggtgcagctgcaacagtcagatcca...ggactggtgaagccctttcagaccctctca ctcaccagtgctgtctgtggattgtccatcaca......accatcaatcaatggtggaac tgaatctggcagcgcccacgaaaggggctggagtggaatgggaagatcagctacgat... ......ggttgcacaaagtttaacccatccctcaat...ggccacacctctatctccaga gtcaca...tccaagggatcattctccctgcagctgagctctgtgactgctgaggacatg gctgtgtattactgctccagagg |
| IMGT000040 | IGHV7-34*01 | aaggtccagctggtgcagtctggggct...gaggtgaagaagcctggggaatccgtgaag gtttcctgcaaggcttctggatacaccttc............actgaatatgctatgaac tgggtgtgacagacccctggaaaaggactcaagtggatgggatggattaacacaaat... ...actgggaagcccacgtatgcctccggcttctca...gaacgatttgtcttctccatg gacgcc...tctgtcagcacggcctatctacagatcagcagcctgaagtctgaggacaca gccacatattactgtgcaagact |
| NW 001876796 | IGHV7-34*02 | aaggtccagctggtgcagtctggggct...gaggtgaagaagcctggggaatccgtgaag gtttcctgcaaggcttctggatacaccttc............actgaatatgctatgaac tgggtgtgacagacccctggaaaaggactcaagtggatgggatggattaacacaaat... ...actgggaagcccacgtatgcctccggcttctca...gaacgatttgtcttctccatg gacgcc...tctgtcagcacggcctatctacagatcagcagcctgaagtctgaggacaca gacacatattactgtgcaagact |
| IMGT000040 | IGHV7-34D*01 | aaggtccagctggtgcagtctggggct...gaggtgaagaagcctggggaatccgtgaag gtttcctgcaaggcttctggatacaccttc............actgaatatgctatgaac tgggtgtgacagacccctggaaaaggactcaagtggatgggatggattaacacaaat... ...actgggaagcccacgtatgcctccggcttctca...gaacgatttgtcttctccatg gacgcc...tctgtcagcacggcctatctacagatcagcagcctgaagtctgaggacaca gccacatattactgtgcaagact |

**
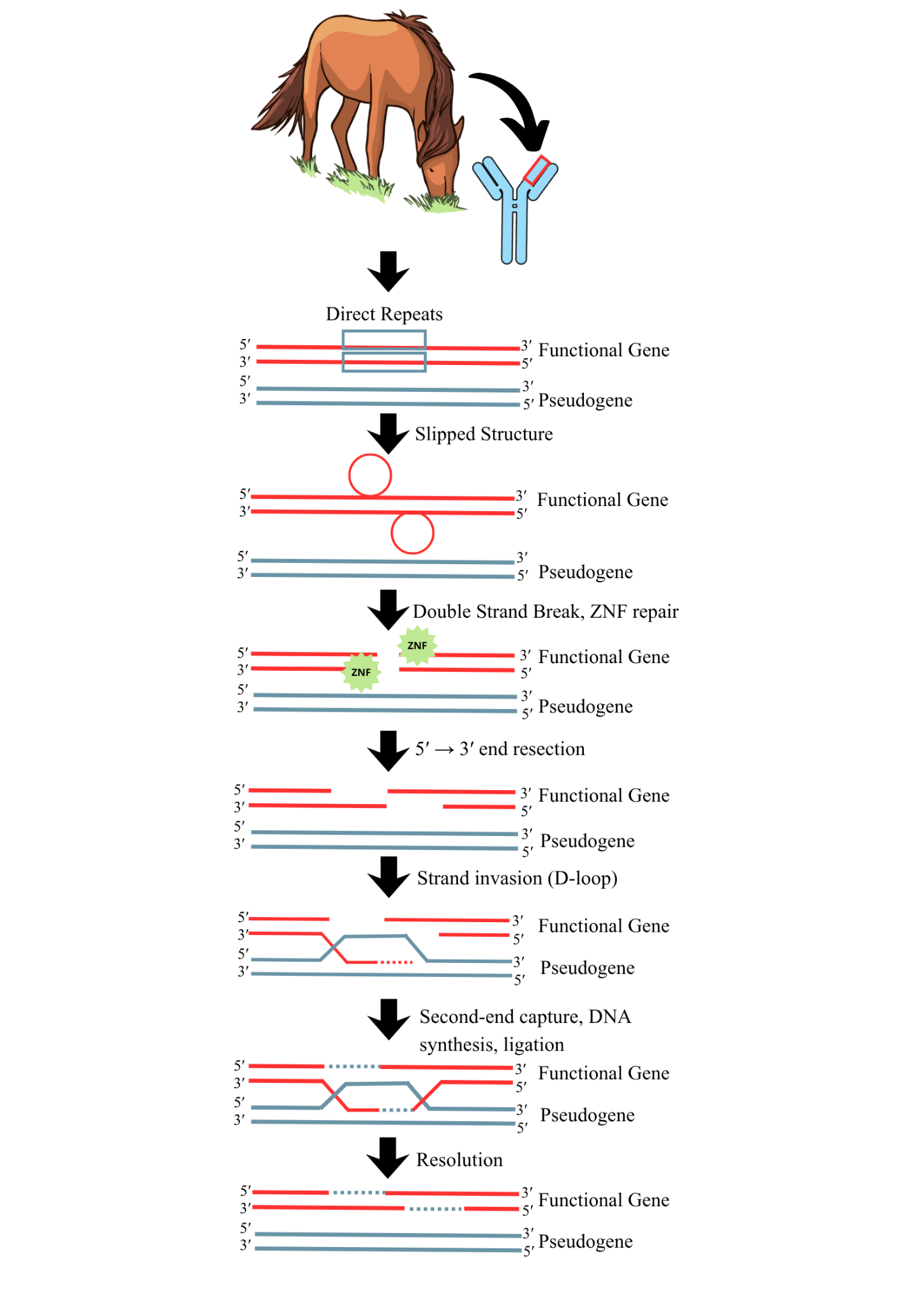
**

**Supplementary Figure 6:** Suggested mechanism for gene conversion events in the horse IGHV region. Directed repeats may be a preferential region for the formation of slipped structures, which can lead to double-strand breaks. These breaks can be repaired by zinc finger (ZNF) proteins, resulting in 5' to 3' end resection, strand invasion and the formation of a displacement loop (D-loop), leading to homologous recombination repair and gene conversion events.
